# Effects of Dietary or Supplementary Micronutrients on Sex Hormones and IGF-1 in Middle and Older Age: A Systematic Review and Meta-Analysis

**DOI:** 10.3390/nu12051457

**Published:** 2020-05-18

**Authors:** Ryan Janjuha, Diane Bunn, Richard Hayhoe, Lee Hooper, Asmaa Abdelhamid, Shaan Mahmood, Joseph Hayden-Case, Will Appleyard, Sophie Morris, Ailsa Welch

**Affiliations:** 1 Norwich Medical School, University of East Anglia, Norwich Research Park, Norwich, Norfolk NR4 7TJ, UK; ryan@janjuha.co.uk (R.J.); r.hayhoe@uea.ac.uk (R.H.); l.hooper@uea.ac.uk (L.H.); asmaa.abdelhamid@uea.ac.uk (A.A.); shaan.mahmood@nuh.nhs.uk (S.M.); joehaydencase@outlook.com (J.H.-C.); will.appleyard@outlook.com (W.A.); sophierose64@gmail.com (S.M.); 2 School of Health Sciences, University of East Anglia, Norwich Research Park, Norwich, Norfolk NR4 7TJ, UK; d.bunn@uea.ac.uk

**Keywords:** micronutrients, sarcopenia, sex hormones, insulin-like growth factor 1, meta-analysis, randomized controlled trials

## Abstract

Observational research suggests that micronutrients may be protective for sarcopenia, a key health issue during ageing, potentially via effects on hormone synthesis and metabolism. We aimed to carry out a systematic review of RCTs investigating effects of increasing dietary or supplemental micronutrient intake on sex hormones and IGF-1 in individuals aged 45 years or older. We searched MEDLINE, EMBASE and Cochrane databases for RCTs reporting the effects of different micronutrients (vitamins A, C, D, or E; carotenoids; iron; copper; zinc; magnesium; selenium; and potassium) on sex hormones or IGF-1. Of the 26 RCTs identified, nine examined effects of vitamin D, nine of multi-nutrients, four of carotenoids, two of selenium, one of zinc, and one of vitamin E. For IGF-1 increasing vitamin D (MD: −0.53 nmol/L, 95% CI: −1.58, 0.52), multi-nutrients (MD: 0.60 nmol/L, 95% CI −1.12 to 2.33) and carotenoids (MD −1.32 nmol/L; 95% CI −2.76 to 0.11) had no significant effect on circulating concentrations. No significant effects on sex hormones of other micronutrients were found, but data were very limited. All trials had significant methodological limitations making effects of micronutrient supplementation on sex hormones unclear. Further high quality RCTs with physiological doses of micronutrients in people with low baseline intakes or circulating concentrations, using robust methodology, are required to assess effects of supplementation adequately.

## 1. Introduction

Sarcopenia is a major problem, involving loss of skeletal muscle mass and function with age, a process beginning at approximately 40 years in both men and women [1,2,3]. One mechanism for its onset, is the age-related decline in the endocrine system, including the secretion of sex hormones and insulin-like growth hormone-1 (IGF-1) [4]. Recent evidence suggests that certain micronutrients may be protective for sarcopenia, and also important for hormone synthesis and metabolism, particularly during the decrease in endogenous secretion that occurs during aging [4]. This decrease in hormone secretion is also associated with increases in risks of falls, osteoporosis, fractures, cardiovascular disease and all-cause mortality [5,6,7,8,9,10,11,12,13,14,15,16].

The endocrine system decline with age [4] includes a decrease in testosterone concentrations of 0.5%–1% per year in men, and of oestrogen in women, that begins around 30 years of age [17,18]. The decline in testosterone concentrations in men is associated with loss of muscle mass and strength [19,20], and furthermore testosterone/dihydrotestosterone (DHT) supplementation can increase muscle strength [21]. Similarly, oestrogen concentrations, which decline more rapidly during the menopause in women [22], are closely linked to muscle strength [23]. Evidence from randomised controlled trials (RCTs) suggests oestrogen replacement therapy reduces the decline in strength of post-menopausal women [22] via a reduction in ‘FOXO3’ activation and ‘MuRF1’ protein expression [24].

The concentration of other endocrine hormones, (Dehydroepiandrosterone, DHEAS; Sex-Hormone Binding Globulin, SHBG; and Insulin-like Growth Factor-1, IGF-1) are also associated with skeletal muscle [25,26] and may be involved in the aetiology of sarcopenia since circulating concentrations change with age. DHEAS [27], which is converted into the active forms of testosterone and oestrogen, and stimulates production of IGF-1 [18], declines with age and relates to loss of muscle mass and strength [17]. SHBG transports testosterone, oestrogen, and other steroids in the blood, and increases with age thus reducing free testosterone and oestrogen [28,29,30]. The age-related decline in IGF-1 [31] is relevant due to its roles in promoting myoblast proliferation and differentiation, as well as formation of muscle fibres during normal growth, and in response to injury [32]. Alongside improving muscle hypertrophy and strength, IGF-1 also suppresses muscle inflammation and fibrosis, and is associated with skeletal muscle mass and strength [32,33,34,35,36]. Therefore, increasing concentrations of circulating IGF-1 and sex hormones may be potentially beneficial for preventing sarcopenia as well as certain non-communicable diseases and conditions of aging.

Micronutrients are potentially important for sex hormone synthesis and metabolism, particularly during the age-related decline in the endocrine system. Previous research from in-vitro, in-vivo or observational studies found that certain micronutrients including vitamin D [37,38,39,40,41,42,43,44,45,46,47], vitamin E [48,49,50], vitamin A [51], lycopene [52], iron [53], magnesium [54,55,56], selenium [57,58,59,60] or zinc [58,60,61,62,63,64,65,66,67] were associated with either androgen metabolism, testosterone concentrations or SHBG. Associations have also been found between oestrogen and vitamins C, D, E, A, and carotenoids, including lycopene [48,68,69,70,71]. IGF-1 has also been associated with lycopene [72,73,74,75], magnesium [55], selenium [57] or zinc [61,62,76,77], iron/ferritin [78] and copper [79]. These studies indicate the relevance of micronutrients to the endocrine system, although many were in individuals in young adulthood and their effects in older age have been less studied to date.

The mechanisms for the role of micronutrients in synthesis of sex hormones and IGF-1 include the involvement in steroidogenesis, via the involvement of prostaglandins, on the precursors of sex hormones, for vitamins D, E, the carotenoids, zinc and selenium, as well the effects of vitamin C [38,39,40,41,42,43,44,45,46,47,48,49,50,52,60,64,65,68,69,71,79,80,81,82,83,84,85,86], and effects on transporter proteins. Zinc is also an inhibitor of two enzymes, aromatase and 5α-reductase, that are involved in testosterone metabolism [60].

Intake of micronutrients, micronutrient deficiency, as well as protein intake, may be also important in determining the onset of sarcopenia [38,76,77,87,88,89,90,91,92,93,94,95,96]. Recent observational and animal studies found that vitamins C, D, E, and carotenoids and the minerals magnesium, selenium, iron and zinc are relevant to muscle mass and physical performance [76,77,88,89,92,97,98,99]. The mechanisms for the action of these nutrients include involvement in collagen and carnitine synthesis, for vitamin C, activities on skeletal muscle cell differentiation and proliferation, for vitamin D [38] and synthesis of protein and mitochondrial function, for magnesium [38,76,77,87,88,89,90,91,92,93,94,95,96,97].

Further mechanisms for changes in hormones and the musculoskeletal system that occur during aging are the associated increases in low grade circulating inflammatory cytokines and of ROS (Reactive Oxygen Species) [93]. A number of micronutrients act as endogenous antioxidants with the capacity to reduce ROS and circulating inflammatory cytokines. These micronutrients include vitamins A, C, E, the carotenoids [100,101,102,103,104,105], zinc [60,106], magnesium [55] and selenium [57,60]. Therefore, improving intakes or rectifying micronutrient deficiency could potentially affect both the onset of sarcopenia as well as sex hormone and IGF-1 metabolism, via a number of mechanisms, during aging. Improvements in micronutrient intake could be achieved through increased consumption of dietary whole foods, e.g., oranges (rich in vitamin C); or via supplementation of vitamins and minerals, e.g., single component or multivitamin tablets.

We are unaware of any previous systematic reviews that have investigated the importance of micronutrient intakes on sex hormones and IGF-1 in middle and older aged people at risk of sarcopenia. Therefore, given the potential role for micronutrients to influence secretion of these hormones during aging, and the importance of these sex hormones to the aetiology of sarcopenia, we conducted a systematic review (SR) to investigate the effects of dietary or supplemental intake of specified micronutrients and changes in concentration of sex hormones and IGF-1. We included adults aged 45 years or older, since this is the age at which recognisable declines in muscle mass and function, sex hormones and IGF-1 start to occur [107].

## 2. Materials and Methods

The systematic review was conducted in accordance with the Cochrane collaboration guidelines and reported using the PRISMA 2009 checklist [108,109]. The protocol was registered with the International prospective register of systematic reviews (PROSPERO), registration ID: CRD42018098657 [110].

### 2.1. Search Methods

Cochrane Central Register of Controlled Trials (CENTRAL), MEDLINE, and EMBASE were searched to 2nd April 2019 using the ‘Population, intervention, comparators, outcomes, study design’ (PICOS) Framework (see Table 1) without date restrictions. The search strategy can be viewed in Supplementary Table S1, A, but in brief, a MEDLINE search was developed and adapted for EMBASE and Cochrane, and search limiters were used for RCTs as per the ‘Scottish Collegiate Network’ [111].

### 2.2. Eligibility Criteria

We included randomised controlled trials (RCTs) that assessed the effects of additional micronutrients in adults aged at least 45 years on primary outcomes. The primary outcomes were changes or differences in sex hormone concentrations, including: androgens (androstenediol, androstenedione, dihydrotestosterone and testosterone), oestrogens (E2, estradiol, estriol, and estrone), DHEAS, SHBG, and IGF-1 (see Supplementary Table S1, A). Relevant micronutrients were those with known or potential relevance to sex hormone or IGF-1 metabolism and physiology, as well as sarcopenia, and included any one, or combination, of vitamin A [48,69]; vitamin C [76]; vitamin D [38,39,40,41,42,43,44,45,46,47]; vitamin E [48,50]; carotenoids [69]; or the minerals zinc [64,65], magnesium [54], selenium, potassium [76,77], iron/ferritin [78] and copper [79].

Where studies included groups of individuals with varying age ranges, they were included if the mean age was greater than 45 years, or more than 75% of individuals were older than 45 years (in both treatment arms). We included studies that used any micronutrient or hormone extraction method, including biomarkers from blood, plasma, red blood cells, body fat, urine, hair, and nails. We excluded studies where the age of the population was unclear, or where participants stopped, started, or changed, hormonal medication, during a study. Where RCTs examined micronutrients in conjunction with another intervention, e.g., exercise, the study was included only if the comparator group received the same non-dietary intervention. Studies that included participants on active dialysis, or with kidney or liver disease [112], were excluded as these are known to affect endogenous sex hormones and IGF-1 [113,114]. In-vitro studies and studies that used foods as interventions without a reported dose of an eligible nutrient were also excluded. We accepted trials of multivitamins or multi-nutrient studies that included further compounds, other than the micronutrients previously listed. This is because some studies may have used combined vitamins for an intervention and provided information. Studies that fell into this category were reported separately, and were defined as two or more different multi-nutrients in the intervention group compared to placebo. We excluded non-English language papers that we were unable to translate within the research team.

### 2.3. Study Selection

Study selection was conducted in a two-phase process. Screening of titles and abstracts against inclusion/exclusion criteria (Supplementary Table S2, B) was carried out independently by two reviewers (RJ and one of DB, AA, RH, AW, SM, JC, WA). Potential titles and abstracts identified by any reviewer were collected in full text and subsequently assessed against the inclusion/exclusion criteria by at least 2 reviewers. Any disagreements were discussed, a third reviewer was not needed to clarify consensus on eligibility.

### 2.4. Data Extraction

We created and tested a data extraction form for this review (Supplementary Table S3, C). Data extracted included: publication details, aims, objectives, country, setting, design, dates, funding, recruitment method, ethical review, participant demographics, intervention descriptions (including: micronutrient type and extraction methods) and outcomes (method of extraction and hormone type). Data extraction and risk of bias assessment was completed independently in duplicate by RJ and another review team member. We were unable to contact authors on any queries regarding data due to time constraints.

### 2.5. Risk of Bias (Quality Assessment)

Risk of bias assessment was based on the Cochrane Risk of Bias tool (https://handbook-5-1.cochrane.org/chapter_8/table_8_5_a_the_cochrane_collaborations_tool_for_assessing.htm) (Supplementary Table S4, D) [115]. Alongside the typical seven standard domains, we included three further items: ‘hormonal treatment’ bias, where participants may be taking medication(s) that influence sex hormones; ‘sponsorship’ bias, where funding by companies may have influenced the outcome of results; and ‘outcome measurement’ bias, which concerns the differences in accuracy of extraction methods. The Journal of Clinical Endocrinology and Metabolism [116] recommends measurement of sex hormones to be conducted using mass spectrometry, as this conveys the highest degree of accuracy, and lowest bias. Studies that used other (less reliable) methods of hormone extraction, e.g., direct immunoassay [117] or electro chemiluminescent assay [118], were assessed to be at high risk of outcome measurement bias.

### 2.6. Data Synthesis and Statistical Analysis

Meta-analyses were performed only where at least two trials could be combined. We used a random effects model in ‘Review Manager (RevMan) [Computer program] [119]. We produced the forest plots using ‘end data’ for intervention and placebo groups. Outcomes were ‘continuous’ and data for IGF-1, testosterone, and SHBG, reported in non-standard units were converted using an online tool (http://unitslab.com/node/230). Where meta-analysis was not possible or data could not be converted or utilised, results were narratively reported. Some studies reported data as ‘median’ values so could not be included in a meta-analysis, but have been included in some forest plots to help illustrate overall effects. Sensitivity analysis, using fixed-effects meta-analysis, was carried out where at least two trials were combined. Comparison between random and fixed effects meta-analysis allowed small study bias to be assessed [120]. We also intended to use funnel plots to assess small study (publication) bias; but as no meta-analysis included at least 10 studies, this was not useful.

## 3. Results

**Figure 1:**
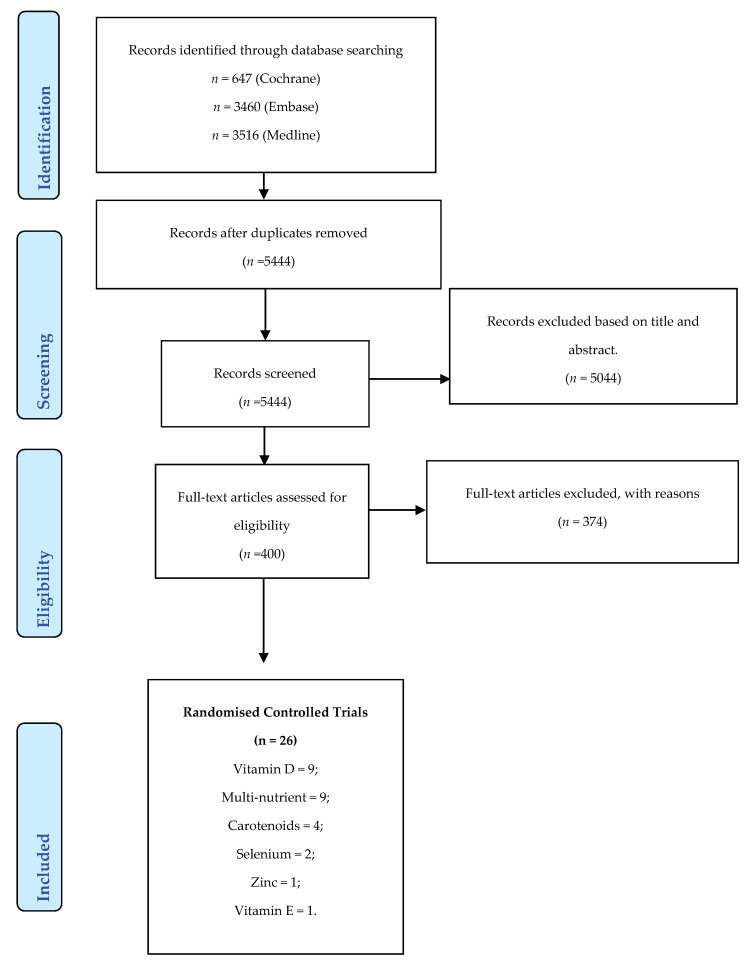
PRISMA Flowchart.

A total of 7623 titles and abstracts were identified from the three separate databases. After the removal of duplicates, 5444 papers remained, of which 5043 were excluded based on title and abstract screening. The remaining 400 studies were assessed in full text, leaving a total of 26 eligible studies. The majority of excluded studies (*n* = 374) were excluded due to the population age or study design. A summary overview of the selection process is provided in Figure 1.

**Figure 2:**
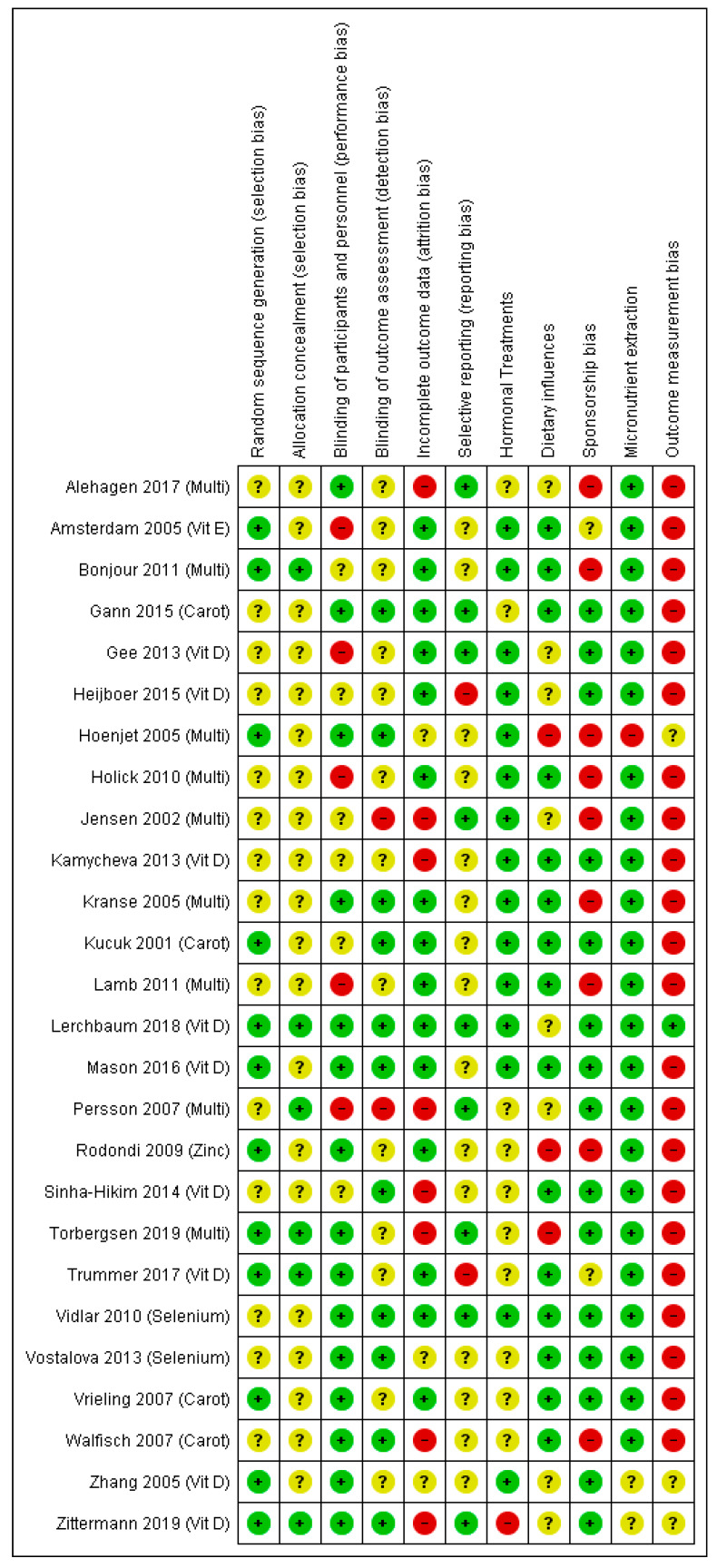
Risk of bias of included RCTs, assessed for each domain and each included trial, based on the Cochrane Risk of Bias tool [120]. +: low risk of bias, ?: unclear risk of bias, -: high risk of bias. Carot, carotenoids; multi, multi-nutrient; vit D, vitamin D; vit E, vitamin E. Please refer to Supplementary Table S5, E to find detailed information on the studies and reference details.

The 26 eligible RCTs examined a range of micronutrients: vitamin D (9, 35%), multi-nutrients (9, 35%), carotenoids (4, 15%), selenium (2, 8%), vitamin E (1, 4%) and zinc (1, 4%). Briefly, a total of 2443 participants were examined. Interventions ranged from 4 weeks to 48 months and participants were mostly males (~64%). Some studies (9, 35%) included individuals who either had a histological diagnosis of prostate cancer or colon cancer, evidence of increasing prostate specific antigen (PSA), or a family history of cancer. Other studies (8, 31%) examined individuals with metabolic syndrome (including obesity) and/or cardiovascular disease. Different races/ethnic groups were also studied, including: Asian, Black, Latino and White. Details of the study characteristics can be found in Supplementary Table S5, E. An overview of the risk of bias for RCTs is shown in Figure 2. We found no trials assessing effects of vitamins A or C, potassium, iron or copper on our outcomes.

### 3.1. Risk of Bias of Included RCTs

Methods of minimising selection bias were poorly reported, with 54% (14/26) and 77% (20/26) of RCT studies being unclear in methodology of ‘randomisation’ and ‘allocation concealment’, respectively (Figure 2). Many studies (58%, or 15/26) minimised performance bias by blinding participants and study personnel, and a smaller proportion (42%, or 11/26) successfully reported blinding of outcomes. Incomplete outcome data was minimised, as was the influence of hormonal treatments on sex hormones (mainly through comprehensive exclusion criteria). Only a small proportion of studies (~12%) advised participants to change their dietary habits in addition to any intervention or placebo. The majority of studies (88%, or 23/26) reported serum blood concentrations, with 12% (3/26) estimating micronutrient intake from dietary assessment questionnaires. Although extraction using serum analysis for micronutrients may pick up coagulants and other trace elements, there appears to be a non-significant variation between plasma and serum values. It is unclear whether the coagulants or trace elements would influence supplemented or non-supplemented cohorts differently. It is worth noting, Olmedilla-Alonso et al. [121] found retinol, gamma- and alpha-tocopherol serum values were positively biased (mean difference of less than: 0.05, 0.01 and 0.7 µmol/L, respectively) when compared to plasma values [121]. However, this is unlikely to influence our results as we only identified one trial with vitamin E within our systematic review. Only 8% (2/26) of studies measured sex hormones using the gold standard recommendation of mass spectrometry [122].

### 3.2. Vitamin D

Nine studies assessed effects of vitamin D on relevant outcomes [116,123,124,125,126,127,128,129,130] but no studies assessed effects on androstenediol, androstenedione, dihydrotestosterone, estriol, or DHEAS. Vitamin D doses varied from 100 IU [130], through 1000 IU [125], 4000 IU [129], 20,000 IU [128] up to 40,000 IU [123], and one was unclear [126]. Baseline vitamin D status was low in some trials [128,129], normal in some [127] and unknown in others [123,124]. Study duration ranged from 6 weeks [130] through 1 year [116,123,125], up to 36 months [129].

#### 3.2.1. Effects of Vitamin D on IGF-1

**Figure 3:**
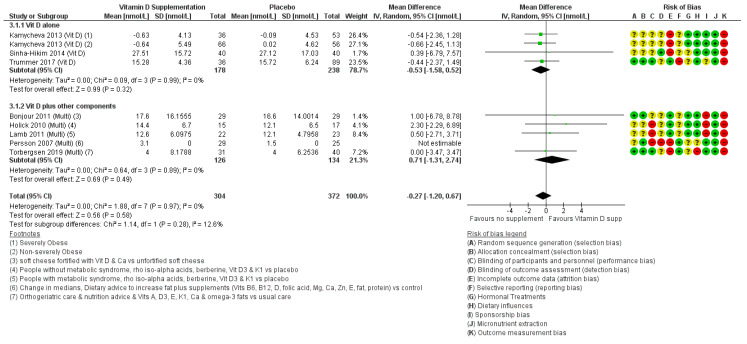
Forest plot assessing effects of increasing vitamin D intake and vitamin D amongst other nutrients, on IGF-1 (nmol/L). Multi, multi-nutrient; vit D, vitamin D. Please refer to Supplementary Table S5, E to find detailed information on the studies and reference details.

Four studies [123,124,125,131] assessed the effects of vitamin D supplementation on IGF-1 over 4 weeks to 12 months. We presented the Kamycheva trial [123] as two groups, severely obese (study participants with >35 kg/m^2^) and non-severely obese (other participants), as results were presented this way in the paper. Meta-analysis demonstrated no significant effects of the intervention (mean difference: −0.53 nmol/L, 95% CI: −1.58, 0.52, 3 RCTs, I2 0%, Figure 3). One trial [124] could not be included in the meta-analysis because it was not possible to convert the units of IGF-1 used (μg/10E06 platelets) to nmol/L. This was a 4-week RCT that confirmed a statistically non-significant mean difference of 0.007 μg/10E06 platelets, *p* = 0.413 between intervention and placebo post intervention. The four included trials randomised 447 participants (mean age: 55.2, 59% males, including dropouts) from Norway [123], USA [124,125] and Austria [131]. Studies used a variety of vitamin D dosages: 400 IU [124] 1000 IU [125], 2800 IU [131] and 40,000 IU [123].

Major sources of bias within these studies included randomisation procedures (sequence generation and allocation concealment) and blinding (Figure 3). Only one trial was at low risk of attrition bias, and no studies used ‘mass spectrometry’ to measure sex hormone concentrations, so all were at high risk of outcome assessment bias. 

The lack of effect of increasing vitamin D on IGF-1 was confirmed in the set of trials which supplemented with vitamin D and other compounds (two or more micronutrients) (Figure 3). Combining all the trials increasing vitamin D (individually or as part of a broader intervention) suggests little or no effect on IGF-1 (MD: −0.27 nmol/L, 95% CI −1.20 to 0.67, I2 0%). This did not differ in sensitivity analysis using fixed-effects meta-analysis (MD: −0.27 nmol/L, 95% CI −1.20 to 0.67, I2 0%). The difference in effect size between fixed- and random-effects meta-analysis suggests that there may be some small study bias present.

The effect of differing baseline vitamin D status, doses and study duration were assessed in sub-grouping. There were no differences between subgroups in any analysis (*p* ≥ 0.85 for all subgroupings, not shown).

#### 3.2.2. Effects of Vitamin D on Testosterone

**Figure 4:**
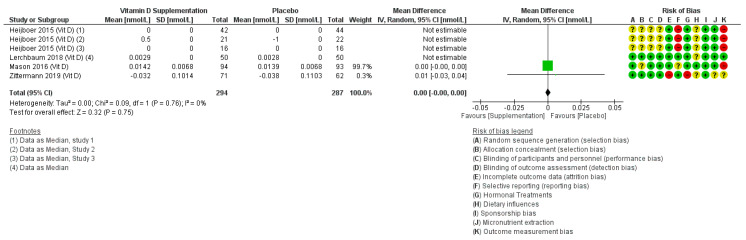
Forest plot assessing effects of increasing vitamin D on free testosterone (nmol/L). Vit D, vitamin D. Please refer to Supplementary Table S5, E to find detailed information on the studies and reference details.

Five trials reported effects of vitamin D on testosterone. They included 754 participants (54% male) from China [126], The Netherlands [127], Austria [128], Germany [129] and USA [116]. All included women were post-menopausal. Only two studies could be combined in meta-analysis, suggesting no effect of vitamin D on free testosterone (MD 0.00, 95% CI −0.00 to 0.00, I2 0%, Figure 4). The effect did not differ in sensitivity analysis using fixed-effects meta-analysis, suggesting a lack of small study bias, although with only two trials this is difficult to assess. The other trials (shown in Figure 4 though not combined in meta-analysis) reported data as medians and interquartile ranges [127,128]. One study did not specify which type of testosterone was measured and did not provide enough data to be included [126].

The two trials that could be combined appeared to have adequate randomisation and blinding, though only one had adequate allocation concealment. Neither was at a low risk of outcome measurement bias.

#### 3.2.3. Effects of Vitamin D on Oestradiol

Two trials assessed effects of vitamin D on oestradiol, but could not be combined in meta-analysis. Individually, neither found a statistically significant effect of supplementation [116,126].

#### 3.2.4. Effects of Vitamin D on SHBG

**Figure 5:**
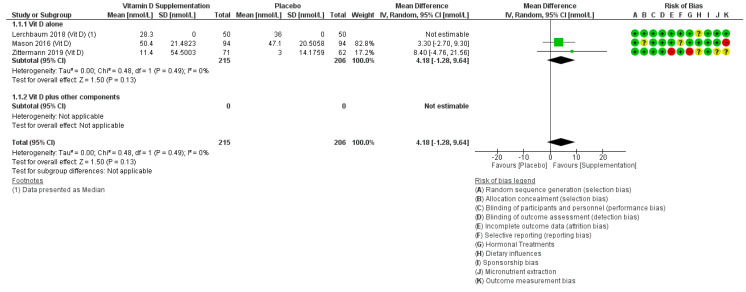
Forest plot showing effects of increasing vitamin D on SHBG (nmol/L). Vit D, vitamin D. Please refer to Supplementary Table S5, E to find detailed information on the studies and reference details.

Three studies reported effects of vitamin D on SHBG, randomising 445 participants (51% male) [116,128,129]. Meta-analysis of two RCTs suggested a small though non-statistically significant increase in SHBG with vitamin D (MD 4.18 nmol/L, 95% CI −1.28 to 9.64, I2 0%, Figure 5), but data from the third trial contradicted this finding. Two of the three trials were at low risk of selection bias, and all were well blinded, and one used a low risk method of outcome assessment (Figure 5).

### 3.3. Multi-Nutrients

Nine studies assessed effects of multi-nutrients (defined as two or more different micronutrients in the intervention group) on relevant outcomes, but no studies assessed effects on androstenediol, androstenedione, E2, estradiol, estriol, estrone, or DHEAS. Those studies that also included vitamin D within the multi-nutrient interventions are also covered in Section 3.2.

#### 3.3.1. Effects of Multi-Nutrient Supplements on IGF-1

**Figure 6:**
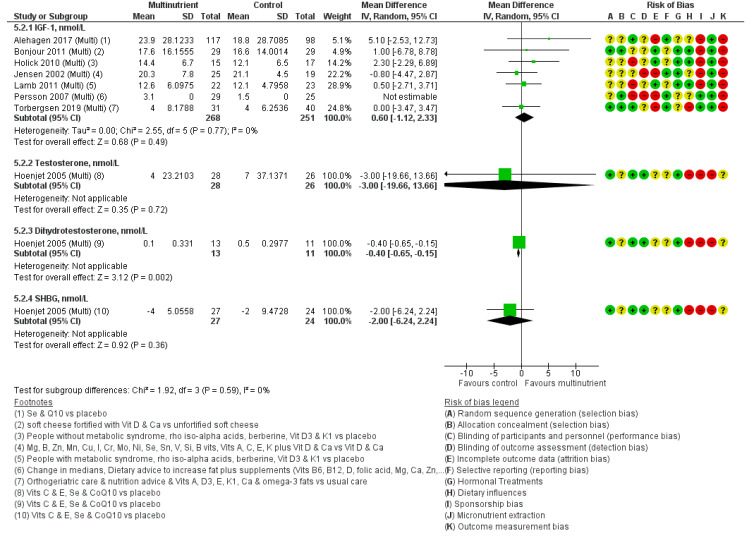
Forest plot assessing the effects of multi-nutrient interventions on sex hormones and IGF-1 (nmol/L). Multi, multi-nutrient. Please refer to Supplementary Table S5, E to find detailed information on the studies and reference details.

Seven of the nine studies identified as multi-nutrient interventions [130,132,133,134,135,136,137] reported on IGF-1, suggesting little or no effect (MD: 0.60 nmol/L, 95% CI −1.12 to 2.33, I2 0%, 519 participants, Figure 6). Effects did not differ in fixed-effects meta-analysis (MD: 0.60 nmol/L, 95% CI −1.12 to 2.33), suggesting that small study bias is not an issue here.

The Jensen et al. study [137] analysed data using a ‘per-protocol’ method which introduced potential bias [138], since the 20% of study participants withdrew. All seven studies measured micronutrient concentrations from blood samples, but none used mass-spectrometry to measure sex hormone concentrations, and many were unclear on selection bias and blinding.

#### 3.3.2. Effects of Multi-Nutrients on Testosterone

Two trials carried out in the Netherlands reported effects of multi-nutrient interventions on testosterone in men with rising levels of prostate-specific antigen (117 males, mean age: 72), and reported opposing findings (Figure 6). We were unable to meta-analyse these findings as Kranse did not provide any measure of variance. However, Hoenjet, 2005 [139] suggested no effect on testosterone concentrations (*p* = 0.28), while Kranse, 2005 [140] (cross-over trial) suggested significant reductions in testosterone but reported different numbers in different places in their paper, so the effect size was unclear (*p* = 0.02). Both trials were at unclear risk of selection bias and low risk of blinding problems.

#### 3.3.3. Effects of Multi-Nutrients on Dihydrotestosterone and SHBG

The same two trials (Kranse, 2005; and Hoenjet, 2005) also assessed the effect of multi-nutrient supplementation on Dihydroteststerone and SHBG (Figure 6), but again Kranse provided no measure of variance and two different effect sizes, so could not be pooled. Multi-nutrient supplementation in Kranse, 2005, reportedly significantly decreased Dihydrotestosterone, but the effect size was unclear (*p* = 0.005), whereas, in Hoenjet 2005, non-significant findings were reported (MD: 0.1 nmol/L, 95% CI −0.1 to 0.2, *p* = 0.72). After supplementation with multi-nutrients, both studies reported non-significant decreases in SHBG.

### 3.4. Carotenoids

Four trials assessed the effects of carotenoids on relevant outcomes, but none assessed the effects on androgens (androstenediol, androstenedione, dihydrotestosterone or testosterone), oestrogens (E2, estradiol, estriol, or estrone), DHEAS or SHBG.

#### Effects of Carotenoids on IGF-1

**Figure 7:**
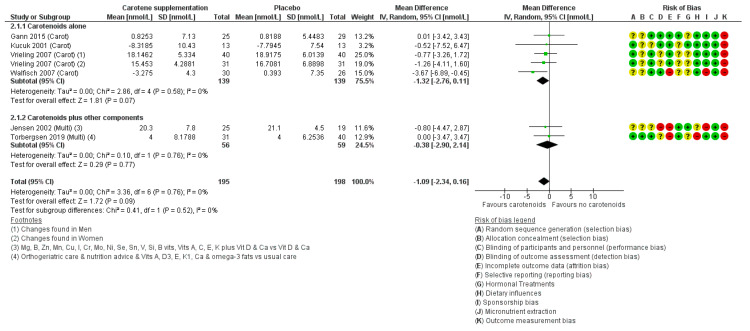
Forest plot assessing effects of increasing carotenoids (lycopene) and carotenoids amongst other nutrients on IGF-1 (nmol/L). Carot, carotenoids; multi, multi-nutrient. Please refer to Supplementary Table S5, E to find detailed information on the studies and reference details.

Four studies [52,80,81,82] examined the effects of lycopene, all using ‘Lyco-O-Mato’ (containing ~15 mg lycopene, plus 1.5 mg phytoene, 1.4 mg phytofluene, 0.4 mg beta-carotene, and 5 mg alpha tocopherol). Meta-analysis of 278 randomised participants (mean age: 63.0, 75% male) showed a non-significant decrease in IGF-1 as a result of the added carotenoids (MD −1.32 nmol/L; 95% CI −2.76 to 0.11, I2 0%, Figure 6). None of the trials were at low risk of selection bias, but two were at low risk from issues around blinding (performance and detection bias), and none used mass-spectrometry to measure hormone concentrations (Figure 7).

Trials of carotenoids as part of multi-nutrient supplementation (Figure 7), confirmed a small non-significant decrease in IGF-1 (MD: −0.39, 95% CI −2.90 to 0.11, I2 0%). These trials also demonstrated significant sources of bias (see earlier).

Overall effects of carotenoids (in either individual or multi-nutrient studies) suggested no important effect of carotenoids on IGF-1 (MD: −1.09, 95% CI −2.34 to 0.16, I2 0%), which did not differ in sensitivity analysis using fixed effects meta-analysis (MD: −1.09, 95% CI −2.34 to 0.16). This suggested minimal small study bias.

### 3.5. Selenium

Two studies assessed effects of selenium on testosterone, but no studies assessed effects on oestrogens (E2, estradiol, estriol, or estrone), DHEAS, SHBG, IGF-1 or androgens other than testosterone.

#### Effects of Selenium on Testosterone

Two studies in the Czech Republic examined the effects of 240 μg of selenium (as selenomethionine) on testosterone. Both intervention and placebo also received 570 mg of silymarin, an extract of milk thistle [141,142]. Both suggested no significant effects on testosterone.

### 3.6. Vitamin E

One study assessed the effects of Vitamin E on DHEAS, but no studies assessed the effects on androgens (androstenediol, androstenedione, dihydrotestosterone and testosterone), oestrogens (E2, estradiol, estriol, and estrone), SHBG, or IGF-1.

#### Effects of Vitamin E on DHEAS

Amsterdam 2005 [143] found that 200 mg vitamin E (as dl-alpha-tocopheryl acetate) over 15 months lead to a significant decrease in DHEAS in the supplemented group (*p* < 0.02) but not the placebo group (*p* > 0.05). The authors concluded there was no overall significant benefit to vitamin E supplementation.

### 3.7. Zinc

One study assessed effects of zinc on IGF-1, but no studies assessed effects on androgens (androstenediol, androstenedione, dihydrotestosterone and testosterone), oestrogens (E2, estradiol, estriol, and estrone), DHEA or SHBG.

#### Effects of Zinc on IGF-1

A Swiss trial by Rodondi, 2009 [61] (*n* = 69, mean age 85, 86% female) reported that supplementation of 30 mg/day of zinc (alongside 15 g whey protein + 5 g amino acids) increased serum IGF-1 over a week compared to protein alone (+48.2% vs. +22.4%, respectively; *p* < 0.027), but there was no statistically significant difference between groups at 4 weeks (+29.2% vs. +45.8%; *p* > 0.05).

## 4. Discussion

We found 26 trials assessing effects of micronutrient supplementation, but no trials assessing effects of vitamins A or C, potassium, iron, or copper, on our outcomes. Data from nine trials suggested that supplementation with vitamin D had little or no effect on IGF-1, with or without other micronutrient compounds. Vitamin D also failed to significantly alter testosterone or oestradiol, and the effects on SHBG and other outcomes were unclear. The multinutrient trials did not suggest statistically significant increases in IGF-1, and the effects on testosterone, dihydrotestosterone and SHBG were unclear. Data were very limited for effects of other micronutrients. Four trials suggested that carotenoids slightly reduce IGF-1 and this was reinforced with the inclusion of other micronutrients (though none of the relationships were statistically significant). Selenium appears to have little effect on testosterone (2 trials), vitamin E had no effect on DHEAS, and zinc had little or no effect on IGF-1 (a single trial each).

Despite our systematic search including a large range of relevant micronutrients and hormones, we only identified studies that investigated effects of vitamin D, multi-nutrients, the carotenoids, selenium, vitamin E, and zinc, on sex hormones and IGF-1. To the best of our knowledge, this is the first systematic review examining the relationship between this range of micronutrients, sex hormones and IGF-1 in people of middle and older age. We conducted the review using established Cochrane methodology [115].

Despite the biochemical, physiological and mechanistic roles of micronutrients for hormone synthesis in older age our review found a paucity of trials and little direct evidence of significant effects of micronutrient supplementation [38,39,40,41,42,43,44,45,46,47,48,49,50,52,60,64,65,68,69,71,79,80,81,82,83,84,85,86]. Since the age-related decline in sex-hormones and IGF-1 not only increases the risk of sarcopenia, but also a number of conditions of aging, including falls, osteoporosis, fractures, cardiovascular disease and all-cause mortality, this is unfortunate [5,6,7,8,9,10,11,12,13,14,15,16].

### Limitations of the Available Data

Whilst we identified 26 RCTs of adults aged at least 45 years that met our eligibility criteria, when grouped by micronutrient and sex-hormone, the number of studies in each category was small (between one and nine studies per nutrient), and many had methodological limitations. A number of the studies had small sample sizes or lack of control for dietary or lifestyle determinants in the intervention and control groups [52,80,82] and one study [137,138] also analysed data using a ‘per-protocol’ method which may have introduced bias elements of bias [138].

For multi-nutrient interventions the composition of the nutrients varied substantially [130,132,133,134,135,136] with some containing more than 20 different micronutrients [132,137], making it difficult to attribute benefit to any specific micronutrient. A number of studies also included additional protein making it difficult to isolate any specific effects of micronutrients from those of protein [61,132,137,144,145]. The baseline nutritional status of participants was not taken into account in a number of studies despite baseline status or deficiency being likely to determine the response to interventions. Some studies also included dietary advice to increase sources of calcium, which may have affected the results of the intervention.

Whilst we found no significant effect of the supplementation of micronutrients on circulating sex hormones and IGF-1, the scale of effects for the few studies that included IGF-1 ranged between mean differences for vitamin D of −0.53 nmol/L (95% CI: −1.58, 0.52), for multi-nutrients of 0.60 nmol/L (95% CI −1.12 to 2.33) and carotenoids of −1.32 nmol/L (95% CI −2.76 to 0.11). This compares with the difference for IGF-1 between age groups 50–54 years to 70–74 years of −3.4 nmol/L [146,147]. Although the effect sizes found with micronutrients and IGF-1 in our analysis were non-significant, and smaller than with age, these differences may have potential importance if found to be significant in future well-designed trials.

## 5. Recommendations for Future Studies

Although our systematic review demonstrated no conclusive effects of the supplementation of micronutrients on sex hormones in middle- and older-aged people, we recommend that larger RCTs are conducted specifically targeting the micronutrients where we found little or no existing research (magnesium, zinc, vitamins A, C and E, iron, copper and potassium). Future RCTs should be of sufficient size and include baseline and follow-up measures of dietary intake (such as with food frequency questionnaires), as well as using blood concentrations of the relevant micronutrients. This would clarify whether micronutrient supplementation is only beneficial to depleted individuals or whether it can provide additional benefit to those with adequate micronutrient status. Direct measurements of micronutrient status have advantages as they are independent of potential reporting bias, are integrated measurements of intake and other physiological and lifestyle influences on status, such as smoking habit, and can be used to determine whether supplementation results in improved micronutrient status [148,149,150]. Furthermore, dosages of micronutrients should be designed to rectify any pre-existing micronutrient deficiency. Additionally, extraction of hormones should be performed using mass spectrometry, and SHBG should be measured to account for changes to free oestrogen and testosterone that may occur during the intervention. Other known lifestyle factors that affect circulating sex hormones and IGF-1, such as smoking habit and BMI should also be recorded [146,147]. An optimal follow-up time has yet to be elucidated but we would recommend a minimum of 6 months, and that endocrine and nutritional measurements be taken at 3 month intervals until the study is complete.

## 6. Conclusions

Effects of micronutrient supplementation on sex hormones and IGF-1 are unclear. Further high quality RCTs with physiological doses of micronutrients in people with low baseline intakes or circulating concentrations, using robust methodology, are required to assess effects adequately.

## Figures and Tables

**Table 1:** PICO Framework for search strategy. See Supplementary Tables (Table S1, A to Table S5, E), for further details.

**P**	Humans, adults only, aged >45 years.
**I**	Micronutrients
**C**	-
**O**	Sex hormones and IGF-1
**S**	Randomised controlled trials (RCTs)

## Supplementary Materials

The following are available online at https://www.mdpi.com/2072-6643/12/5/1457/s1, Table S1, E: Search Strategy Form, Table S2, B: Inclusion/Exclusion Form, Table S3, C: Data Extraction Form, Table S4, D: Systematic review Cochrane Risk of Bias Form, Table S5, E: Table of Characteristics of RCTs (*n* = 26).

## References

[1] (1989). Epidemiologic and methodologic problems in determining nutritional status of older persons. Proceedings of a conference. Albuquerque, New Mexico, October 19–21, 1988. Am. J. Clin. Nutr..

[2] Boss G.R., Seegmiller J.E. (1981). Age-Related Physiological Changes and Their Clinical Significance. West. J. Med..

[3] Marzetti E., Calvani R., Tosato M., Cesari M., Di Bari M., Cherubini A., Collamati A., D’Angelo E., Pahor M., on behalf of the SPRINTT Consortium (2017). Sarcopenia: An overview. Aging Clin. Exp. Res..

[4] Marty E., Liu Y., Samuel A., Or O., Lane J. (2017). A review of sarcopenia: Enhancing awareness of an increasingly prevalent disease. Bone.

[5] Vandenput L., Mellström D., A Laughlin G., Cawthon P.M., A Cauley J., Hoffman A.R., Karlsson M.K., Rosengren B.E., Ljunggren Ö., Nethander M. (2017). Low Testosterone, but Not Estradiol, Is Associated With Incident Falls in Older Men: The International MrOS Study. J. Bone Min. Res..

[6] Roddam A.W., Appleby P., Neale R.E., Dowsett M., Folkerd E., Tipper S., Allen N.E., Key T.J. (2009). Association between endogenous plasma hormone concentrations and fracture risk in men and women: The EPIC-Oxford prospective cohort study. J. Bone Min. Metab..

[7] Khaw K.-T., Dowsett M., Folkerd E., Bingham S., Wareham N., Luben R.N., Welch A., Day N. (2007). Endogenous testosterone and mortality due to all causes, cardiovascular disease, and cancer in men: European prospective investigation into cancer in Norfolk (EPIC-Norfolk) Prospective Population Study. Circulation.

[8] Guadalupe-Grau A., Carnicero J.A., Losa-Reyna J., Tresguerres J., Gomez-Cabrera M.C., Castillo C., Alfaro-Acha A., Rosado-Artalejo C., Rodríguez-Mañas L., Garcia-Garcia F.J. (2017). Endocrinology of Aging From a Muscle Function Point of View: Results from the Toledo Study for Healthy Aging. J. Am. Med. Dir. Assoc..

[9] Clegg D., Hevener A.L., Moreau K.L., Morselli E., Criollo A., Van Pelt R.E., Vieira-Potter V.J. (2017). Sex Hormones and Cardiometabolic Health: Role of Estrogen and Estrogen Receptors. Endocrinology.

[10] Diamanti-Kandarakis E., Dattilo M., Macut Đ., Duntas L.H., Gonos E.S., Goulis D.G., Kanaka-Gantenbein C., Kapetanou M., Koukkou E.G., Lambrinoudaki I. (2017). MECHANISMS IN ENDOCRINOLOGY: Aging and anti-aging: A Combo-Endocrinology overview. Eur. J. Endocrinol..

[11] Horstman A.M., Dillon E.L., Urban R.J., Sheffield-Moore M. (2012). The role of androgens and estrogens on healthy aging and longevity. J. Gerontol. A Biol. Sci. Med. Sci..

[12] Yeap B.B., Alfonso H., Chubb S.A.P., Center J.R., Beilin J., Hankey G.J., Almeida O.P., Golledge J., Norman P., Flicker L. (2020). U-Shaped Association of Plasma Testosterone, and no Association of Plasma Estradiol, with Incidence of Fractures in Men. J. Clin. Endocrinol. Metab..

[13] Moreau K.L., Babcock M.C., Hildreth K.L. (2020). Sex differences in vascular aging in response to testosterone. Biol. Sex Differ..

[14] Karlamangla A.S., Burnett-Bowie S.M., Crandall C.J. (2018). Bone Health during the Menopause Transition and Beyond. Obstet. Gynecol. Clin. N. Am..

[15] Hidayat K., Du X., Shi B.M. (2018). Sex hormone-binding globulin and risk of fracture in older adults: Systematic review and meta-analysis of observational studies. Osteoporos Int..

[16] Brundle C., Heaven A., Brown L., Teale E., Young J., West R., Clegg A. (2019). Convergent validity of the electronic frailty index. Age Ageing.

[17] Morley J.E. (2017). Hormones and Sarcopenia. Curr. Pharm. Des..

[18] Maggio M., Lauretani F., Ceda G.P. (2013). Sex hormones and sarcopenia in older persons. Curr. Opin. Clin. Nutr. Metab. Care.

[19] Baumgartner R.N., Waters D.L., Gallagher D., Morley J.E., Garry P.J. (1999). Predictors of skeletal muscle mass in elderly men and women. Mech. Ageing Dev..

[20] Mouser J.G., Loprinzi P.D., Loenneke J.P. (2016). The association between physiologic testosterone levels, lean mass, and fat mass in a nationally representative sample of men in the United States. Steroids.

[21] Ottenbacher K.J., Ottenbacher M.E., Ottenbacher A.J., Acha A.A., Ostir G.V. (2006). Androgen Treatment and Muscle Strength in Elderly Males: A Meta-Analysis. J. Am. Geriatr. Soc..

[22] Tiidus P.M. (2011). Benefits of Estrogen Replacement for Skeletal Muscle Mass and Function in Post-Menopausal Females: Evidence from Human and Animal Studies. Eurasian J. Med..

[23] Samson M.M., Meeuwsen I.B., Crowe A., Dessens J.A., Duursma S.A., Verhaar H.J. (2000). Relationships between physical performance measures, age, height and body weight in healthy adults. Age Ageing.

[24] Park Y.-M., Keller A.C., Runchey S.S., Miller B.F., Kohrt W.M., Van Pelt R.E., Kang C., Jankowski C.M., Moreau K.L. (2019). Acute estradiol treatment reduces skeletal muscle protein breakdown markers in early- but not late-postmenopausal women. Steroids.

[25] Velders M., Diel P. (2013). How sex hormones promote skeletal muscle regeneration. Sports Med..

[26] Taekema D.G., Ling C.H., Blauw G.J., Meskers C.G., Westendorp R.G.J., De Craen A.J.M., Maier A.B. (2011). Circulating levels of IGF1 are associated with muscle strength in middle-aged- and oldest-old women. Eur. J. Endocrinol..

[27] Warner M., Gustafsson J.A. (2015). DHEA-a precursor of ERbeta ligands. J. Steroid Biochem. Mol. Biol..

[28] Selby C. (1990). Sex hormone binding globulin: Origin, function and clinical significance. Ann. Clin. Biochem..

[29] Liu P.Y., Beilin J., Nguyen T.V., Center J.R., Meier C., Leedman P.J., Seibel M., A Eisman J., Handelsman D.J. (2007). Age-Related Changes in Serum Testosterone and Sex Hormone Binding Globulin in Australian Men: Longitudinal Analyses of Two Geographically Separate Regional Cohorts. J. Clin. Endocrinol. Metab..

[30] Longcope C., Goldfield S.R., Brambilla D.J., McKinlay J. (1990). Androgens, estrogens, and sex hormone-binding globulin in middle-aged men. J. Clin. Endocrinol. Metab..

[31] Pang A.L.-Y., Chan W.-Y., Coleman W.B., Tsongalis G.J. (2010). Chapter 22-Molecular Basis of Diseases of the Endocrine System. Essential Concepts in Molecular Pathology.

[32] Philippou A., Barton E.R. (2014). Optimizing IGF-I for skeletal muscle therapeutics. Growth Horm. IGF Res..

[33] Secco M., Bueno C., Vieira N.M., Almeida C., Pelatti M., Zucconi E., Bartolini P., Vainzof M., Miyabara E., Okamoto O.K. (2013). Systemic delivery of human mesenchymal stromal cells combined with IGF-1 enhances muscle functional recovery in LAMA2 dy/2j dystrophic mice. Stem Cell Rev..

[34] Rybalko V.Y., Pham C.B., Hsieh P.-L., Hammers D.W., Merscham-Banda M., Suggs L.J., Farrar R.P. (2015). Controlled delivery of SDF-1alpha and IGF-1: CXCR4(+) cell recruitment and functional skeletal muscle recovery. Biomater. Sci..

[35] Bucci L., Yani S.L., Fabbri C., Bijlsma A.Y., Maier A.B., Meskers C.G., Narici M., Jones D.A., McPhee J.S., Seppet E. (2013). Circulating levels of adipokines and IGF-1 are associated with skeletal muscle strength of young and old healthy subjects. Biogerontology.

[36] Mohamad M.I., Khater M.S. (2015). Evaluation of insulin like growth factor-1 (IGF-1) level and its impact on muscle and bone mineral density in frail elderly male. Arch. Gerontol. Geriatr..

[37] Harrison P. Low Vitamin D Tied to Testosterone Dip in Healthy Men 2015 [Vitamin D and Testosterone]. https://www.medscape.com/viewarticle/845483.

[38] Ceglia L., Harris S.S. (2013). Vitamin D and its role in skeletal muscle. Calcif Tissue Int..

[39] Azadi-Yazdi M., Nadjarzadeh A., Khosravi-Boroujeni H., Salehi-Abargouei A. (2017). The Effect of Vitamin D Supplementation on the Androgenic Profile in Patients with Polycystic Ovary Syndrome: A Systematic Review and Meta-Analysis of Clinical Trials. Horm. Metab. Res..

[40] Zhao D., Ouyang P., De Boer I.H., Lutsey P.L., Farag Y.M., Guallar E., Siscovick D.S., Post W.S., Kalyani R.R., Billups K.L. (2017). Serum vitamin D and sex hormones levels in men and women: The Multi-Ethnic Study of Atherosclerosis (MESA). Maturitas.

[41] Rafiq R., Van Schoor N., Sohl E., Zillikens M., Oosterwerff M., Schaap L., Lips P., De Jongh R. (2016). Associations of vitamin D status and vitamin D-related polymorphisms with sex hormones in older men. J. Steroid Biochem. Mol. Boil..

[42] Chin K.Y., Ima-Nirwana S., Wan Ngah W.Z. (2015). Vitamin D is significantly associated with total testosterone and sex hormone-binding globulin in Malaysian men. Aging Male.

[43] Lerchbaum E., Pilz S., Trummer C., Rabe T., Schenk M., Heijboer A.C., Obermayer-Pietsch B. (2014). Serum vitamin D levels and hypogonadism in men. Andrology.

[44] Anic G.M., Albanes D., Rohrmann S., Kanarek N., Nelson W.G., Bradwin G., Rifai N., McGlynn K.A., Platz E.A., Mondul A.M. (2016). Association between serum 25-hydroxyvitamin D and serum sex steroid hormones among men in NHANES. Clin. Endocrinol..

[45] Chang E.M., Kim Y.S., Won H.J., Yoon T.K., Lee W.S. (2014). Association between Sex Steroids, Ovarian Reserve, and Vitamin D Levels in Healthy Nonobese Women. J. Clin. Endocrinol. Metab..

[46] Jorde R., Grimnes G., Hutchinson M.S., Kjaergaard M., Kamycheva E., Svartberg J. (2013). Supplementation with vitamin D does not increase serum testosterone levels in healthy males. Horm. Metab. Res..

[47] Hammoud A.O., Wayne Meikle A., Matthew Peterson C., Stanford J., Gibson M., Carrell D.T. (2012). Association of 25-hydroxy-vitamin D levels with semen and hormonal parameters. Asian J. Androl..

[48] Mumford S.L., Browne R.W., Schliep K.C., Schmelzer J., Plowden T.C., A Michels K., Sjaarda L., Zarek S.M., Perkins N., Messer L. (2016). Serum Antioxidants Are Associated with Serum Reproductive Hormones and Ovulation among Healthy Women. J. Nutr..

[49] Barella L., Rota C., Stocklin E., Rimbach G. (2004). Alpha-tocopherol affects androgen metabolism in male rat. Ann. New York Acad. Sci..

[50] Hartman T.J., Dorgan J.F., Woodson K., Virtamo J., A Tangrea J., Heinonen O.P., Taylor P.R., Barrett M.J., Albanes D. (2001). Effects of long-term alpha-tocopherol supplementation on serum hormones in older men. Prostate.

[51] Hogarth C.A., Griswold M.D. (2010). The key role of vitamin A in spermatogenesis. J. Clin. Investig..

[52] Kucuk O., Sarkar F.H., Sakr W., Djuric Z., Pollak M.N., Khachik F., Li Y.W., Banerjee M., Grignon D., Bertram J.S. (2001). Phase II randomized clinical trial of lycopene supplementation before radical prostatectomy. Cancer Epidemiol. Biomark. Prev..

[53] Rotter I., Kosik-Bogacka D.I., Dolegowska B., Safranow K., Kuczynska M., Laszczynska M. (2016). Analysis of the relationship between the blood concentration of several metals, macro- and micronutrients and endocrine disorders associated with male aging. Environ. Geochem. Health.

[54] Rotter I., Kosik-Bogacka D., Dolegowska B., Safranow K., Karakiewicz B., Laszczynska M. (2015). Relationship between serum magnesium concentration and metabolic and hormonal disorders in middle-aged and older men. Magnes. Res..

[55] Maggio M., Ceda G., Lauretani F., Cattabiani C., Avantaggiato E., Morganti S., Ablondi F., Bandinelli S., Dominguez L.-J., Barbagallo M. (2011). Magnesium and anabolic hormones in older men. Int. J. Androl..

[56] Cinar V., Polat Y., Baltaci A.K., Mogulkoc R. (2011). Effects of magnesium supplementation on testosterone levels of athletes and sedentary subjects at rest and after exhaustion. Biol. Trace Elem. Res..

[57] Maggio M., Ceda G., Lauretani F., Bandinelli S., Dall’Aglio E., Guralnik J.M., Paolisso G., Semba R.D., Nouvenne A., Borghi L. (2010). Association of plasma selenium concentrations with total IGF-1 among older community-dwelling adults: The InCHIANTI study. Clin. Nutr..

[58] Oluboyo A., Adijeh R.U., Onyenekwe C.C., O Oluboyo B., Mbaeri T.C., Odiegwu C.N., O Chukwuma G., Onwuasoanya U.F. (2012). Relationship between serum levels of testosterone, zinc and selenium in infertile males attending fertility clinic in Nnewi, south east Nigeria. Afr. J. Med. Med. Sci..

[59] Hawkes W.C., Turek P.J. (2001). Effects of dietary selenium on sperm motility in healthy men. J. Androl..

[60] Darago A., Klimczak M., Stragierowicz J., Stasikowska-Kanicka O., Kilanowicz A. (2020). The Effect of Zinc, Selenium, and Their Combined Supplementation on Androgen Receptor Protein Expression in the Prostate Lobes and Serum Steroid Hormone Concentrations of Wistar Rats. Nutrients.

[61] Rodondi A., Ammann P., Ghilardi-Beuret S., Rizzoli R. (2009). Zinc increases the effects of essential amino acids-whey protein supplements in frail elderly. J. Nutr. Health Aging.

[62] Blostein-Fujii A., DiSilvestro R.A., Frid D., Katz C., Malarkey W. (1997). Short-term zinc supplementation in women with non-insulin-dependent diabetes mellitus: Effects on plasma 5’-nucleotidase activities, insulin-like growth factor I concentrations, and lipoprotein oxidation rates in vitro. Am. J. Clin. Nutr..

[63] Vivoli G., Fantuzzi G., Bergomi M., Tonelli E., Gatto M., Zanetti F., Del Dot M. (1990). Relationship between zinc in serum and hair and some hormones during sexual maturation in humans. Sci. Total Environ..

[64] Shafiei Neek L., Gaeini A.A., Choobineh S. (2011). Effect of zinc and selenium supplementation on serum testosterone and plasma lactate in cyclist after an exhaustive exercise bout. Biol. Trace Elem. Res..

[65] Kilic M., Baltaci A.K., Gunay M., Gokbel H., Okudan N., Cicioglu I. (2006). The effect of exhaustion exercise on thyroid hormones and testosterone levels of elite athletes receiving oral zinc. Neuro Endocrinol. Lett..

[66] Ebisch I.M., Thomas C.M., Peters W.H., Braat D.D., Steegers-Theunissen R.P. (2007). The importance of folate, zinc and antioxidants in the pathogenesis and prevention of subfertility. Hum. Reprod. Update.

[67] Ebisch I.M.W., Pierik F.H., De Jong F.H., Thomas C.M.G., Steegers-Theunissen R.P.M. (2006). Does folic acid and zinc sulphate intervention affect endocrine parameters and sperm characteristics in men?. Int. J. Androl..

[68] Vihtamaki T., Parantainen J., Koivisto A.M., Metsa-Ketela T., Tuimala R. (2002). Oral ascorbic acid increases plasma oestradiol during postmenopausal hormone replacement therapy. Maturitas.

[69] Maggio M., De Vita F., Lauretani F., Bandinelli S., Semba R.D., Bartali B., Cherubini A., Cappola A.R., Ceda G., Ferrucci L. (2015). Relationship between Carotenoids, Retinol, and Estradiol Levels in Older Women. Nutrients.

[70] Lerchbaum E. (2014). Vitamin D and menopause—A narrative review. Maturitas.

[71] Lerchbaum E., Obermayer-Pietsch B. (2012). Vitamin D and fertility: A systematic review. Eur. J. Endocrinol..

[72] Karas M., Amir H., Fishman D., Danilenko M., Segal S., Nahum A., Koifmann A., Giat Y., Levy J., Sharoni Y. (2000). Lycopene interferes with cell cycle progression and insulin-like growth factor I signaling in mammary cancer cells. Nutr. Cancer.

[73] Liu C., Lian F., Smith D.E., Russell R.M., Wang X.D. (2003). Lycopene supplementation inhibits lung squamous metaplasia and induces apoptosis via up-regulating insulin-like growth factor-binding protein 3 in cigarette smoke-exposed ferrets. Cancer Res..

[74] Hirsch K., Atzmon A., Danilenko M., Levy J., Sharoni Y. (2007). Lycopene and other carotenoids inhibit estrogenic activity of 17beta-estradiol and genistein in cancer cells. Breast Cancer Res. Treat..

[75] Kanagaraj P., Vijayababu M.R., Ravisankar B., Anbalagan J., Aruldhas M.M., Arunakaran J. (2007). Effect of lycopene on insulin-like growth factor-I, IGF binding protein-3 and IGF type-I receptor in prostate cancer cells. J. Cancer Res. Clin. Oncol..

[76] Welch A.A. (2014). Nutritional influences on age-related skeletal muscle loss. Proc. Nutr. Soc..

[77] Van Dronkelaar C., van Velzen A., Abdelrazek M., van der Steen A., Weijs P.J.M., Tieland M. (2018). Minerals and Sarcopenia; The Role of Calcium, Iron, Magnesium, Phosphorus, Potassium, Selenium, Sodium, and Zinc on Muscle Mass, Muscle Strength, and Physical Performance in Older Adults: A Systematic Review. J. Am. Med. Dir. Assoc..

[78] Liu Z., Ye F., Zhang H., Gao Y., Tan A., Zhang S., Xiao Q., Zhang B., Huang L., Ye B. (2013). The association between the levels of serum ferritin and sex hormones in a large scale of Chinese male population. PLoS ONE.

[79] Aihara K., Nishi Y., Hatano S., Kihara M., Ohta M., Sakoda K., Uozumi T., Usui T. (1985). Zinc, copper, manganese, and selenium metabolism in patients with human growth hormone deficiency or acromegaly. J. Pediatr. Gastroenterol. Nutr..

[80] Walfisch S., Walfisch Y., Kirilov E., Linde N., Mnitentag H., Agbaria R., Sharoni Y., Levy J. (2007). Tomato lycopene extract supplementation decreases insulin-like growth factor-I levels in colon cancer patients. Eur. J. Cancer Prev..

[81] Vrieling A., Voskuil D.W., Bonfrer J.M., Korse C.M., Van Doorn J., Cats A., Depla A.C., Timmer R., Witteman B.J., E Van Leeuwen F. (2007). Lycopene supplementation elevates circulating insulin-like growth factor binding protein-1 and -2 concentrations in persons at greater risk of colorectal cancer. Am. J. Clin. Nutr..

[82] Gann P.H., Deaton R.J., Rueter E.E., Van Breemen R.B., Nonn L., Macias V., Han M., Ananthanarayanan V. (2015). A Phase II Randomized Trial of Lycopene-Rich Tomato Extract Among Men with High-Grade Prostatic Intraepithelial Neoplasia. Nutr. Cancer.

[83] Darago A., Sapota A., Matych J., Nasiadek M., Skrzypinska-Gawrysiak M., Kilanowicz A. (2011). The correlation between zinc and insulin-like growth factor 1 (IGF-1), its binding protein (IGFBP-3) and prostate-specific antigen (PSA) in prostate cancer. Clin. Chem. Lab. Med..

[84] Lerchbaum E., Trummer C., Schwetz V., Pachernegg O., Heijboer A.C., Pilz S., Obermayer-Pietsch B. (2017). Vitamin D and Testosterone in Healthy Men: A Randomized Controlled Trial. J. Clin. Endocrinol. Metab..

[85] Lerchbaum E., Rabe T. (2014). Vitamin D and female fertility. Curr. Opin. Obstet. Gynecol..

[86] Lundqvist J., Norlin M., Wikvall K. (2010). 1alpha,25-Dihydroxyvitamin D3 affects hormone production and expression of steroidogenic enzymes in human adrenocortical NCI-H295R cells. Biochim. Biophys. Acta.

[87] Sayer A.A. (2010). Sarcopenia. BMJ.

[88] Hayhoe R.P.G., Lentjes M.A.H., Mulligan A.A., Luben R.N., Khaw K.T., Welch A.A. (2019). Cross-sectional associations of dietary and circulating magnesium with skeletal muscle mass in the EPIC-Norfolk cohort. Clin. Nutr..

[89] Welch A.A., Jennings A., Kelaiditi E., Skinner J., Steves C.J. (2020). Cross-sectional associations between dietary antioxidant vitamins C,E and carotenoid intakes and sarcopenic indices in women aged 18–79 years. Calcif. Tissue Int..

[90] Cameron D., Welch A.A., Adelnia F., Bergeron C.M., Reiter D.A., Dominguez L.J., Ferrucci L. (2019). Age and function are more closely associated with intracellular magnesium as assessed by 31P Magnetic Resonance Spectroscopy, than with serum mangesium. Front. Physiol..

[91] Landi F., Camprubi-Robles M., E Bear D., Cederholm T., Malafarina V., Welch A.A., Cruz-Jentoft A.J., Landi F. (2019). Muscle loss: The new malnutrition challenge in clinical practice. Clin. Nutr..

[92] Welch A.A., Skinner J., Hickson M. (2017). Dietary Magnesium May Be Protective for Aging of Bone and Skeletal Muscle in Middle and Younger Older Age Men and Women: Cross-Sectional Findings from the UK Biobank Cohort. Nutrients.

[93] Welch A.A., Kelaiditi E., Jennings A., Steves C.J., Spector T.D., MacGregor A. (2016). Dietary Magnesium Is Positively Associated With Skeletal Muscle Power and Indices of Muscle Mass and May Attenuate the Association Between Circulating C-Reactive Protein and Muscle Mass in Women. J. Bone Min. Res..

[94] Semba R.D., Lauretani F., Ferrucci L. (2007). Carotenoids as protection against sarcopenia in older adults. Arch. Biochem. Biophys..

[95] Cermak N.M., Res P.T., de Groot L.C., Saris W.H., van Loon L.J. (2012). Protein supplementation augments the adaptive response of skeletal muscle to resistance-type exercise training: A meta-analysis. Am. J. Clin. Nutr..

[96] Hickson M. (2015). Nutritional interventions in sarcopenia: A critical review. Proc. Nutr. Soc..

[97] Chung E., Mo H., Wang S., Zu Y., Elfakhani M., Rios S.R., Chyu M.-C., Yang R.-S., Shen C.-L. (2018). Potential roles of vitamin E in age-related changes in skeletal muscle health. Nutr. Res..

[98] Takisawa S., Funakoshi T., Yatsu T., Nagata K., Aigaki T., Machida S., Ishigami A. (2019). Vitamin C deficiency causes muscle atrophy and a deterioration in physical performance. Sci. Rep..

[99] Demirbag R., Yilmaz R., Erel O. (2005). The association of total antioxidant capacity with sex hormones. Scand Cardiovasc. J..

[100] Van Poppel G., Goldbohm R.A. (1995). Epidemiologic evidence for beta-carotene and cancer prevention. Am. J. Clin. Nutr..

[101] Sharoni Y., Danilenko M., Dubi N., Ben-Dor A., Levy J. (2004). Carotenoids and transcription. Arch. Biochem. Biophys..

[102] Davison G.W., Ashton T., George L., Young I.S., McEneny J., Davies B., Jackson S.K., Peters J.R., Bailey D.M. (2008). Molecular detection of exercise-induced free radicals following ascorbate prophylaxis in type 1 diabetes mellitus: A randomised controlled trial. Diabetologia.

[103] Alessio H.M., Goldfarb A.H., Cao G. (1997). Exercise-induced oxidative stress before and after vitamin C supplementation. Int. J. Sport Nutr..

[104] Rokitzki L., Logemann E., Huber G., Keck E., Keul J. (1994). alpha-Tocopherol supplementation in racing cyclists during extreme endurance training. Int. J. Sport Nutr..

[105] McAnulty S.R., McAnulty L.S., Nieman D.C., Morrow J.D., Shooter L.A., Holmes S., Heward C., Henson D.A. (2005). Effect of alpha-tocopherol supplementation on plasma homocysteine and oxidative stress in highly trained athletes before and after exhaustive exercise. J. Nutr. Biochem..

[106] De Oliveira Kde J., Donangelo C.M., de Oliveira A.V., da Silveira C.L., Koury J.C. (2009). Effect of zinc supplementation on the antioxidant, copper, and iron status of physically active adolescents. Cell Biochem. Funct..

[107] Landi F., Calvani R., Tosato M., Martone A.M., Fusco D., Sisto A., Ortolani E., Savera G., Salini S., Marzetti E. (2017). Age-Related Variations of Muscle Mass, Strength, and Physical Performance in Community-Dwellers: Results From the Milan EXPO Survey. J. Am. Med. Dir. Assoc..

[108] Reviews C.S. Cochrane Handbook for Systematic Reviews of Interventions. http://community.cochrane.org/handbook.

[109] Moher D., Liberati A., Tetzlaff J., Altman D.G., Group T.P. (2009). Preferred reporting items for sytematic reviews and meta-analyses: The PRISMA statement. PLoS Med..

[110] PRISMA (2009). The PRISMA Checklist: Prisma-statement.org. http://prisma-statement.org/documents/PRISMA%202009%20checklist.pdf.

[111] Network SIG SIGN 50: A Guideline Developers Handbook 2019. https://www.sign.ac.uk/sign-50.

[112] Schmidt A., Luger A., Hörl W.H. (2002). Sexual hormone abnormalities in male patients with renal failure. Nephrol. Dial. Transplant..

[113] Moller S., Becker U. (1992). Insulin-like growth factor 1 and growth hormone in chronic liver disease. Dig. Dis..

[114] Oh Y. (2012). The insulin-like growth factor system in chronic kidney disease: Pathophysiology and therapeutic opportunities. Kidney Res. Clin. Pract..

[115] Cochrane Cochrane Handbook 2017. http://handbook-5-1.cochrane.org/.

[116] Handelsman D.J., Wartofsky L. (2013). Requirement for Mass Spectrometry Sex Steroid Assays in the Journal of Clinical Endocrinology and Metabolism. J. Clin. Endocrinol. Metab..

[117] Stanczyk F.Z., Cho M.M., Endres D.B., Morrison J.L., Patel S., Paulson R.J. (2003). Limitations of direct estradiol and testosterone immunoassay kits. Steroids.

[118] Vieira J.G.H., Nakamura O.H., Ferrer C.M., Tachibana T.T., Endo M.H.K., Carvalho V.M. (2008). The importance of methodology in serum testosterone measurement: Comparison between a direct immunoassay and a method based on high performance liquid chromatography and tandem mass spectrometry (HPLC/MS-MS). Arq. Bras. Endocrinol. Metabol..

[119] Cochran (2014). Review Manager 5 (ReVMan5) [Computer Program]. Version 5.3: Copenhagen: The Nordic Cochrane Centre, The Cochran Collaboration. http://Community.cochrane.org/.

[120] Page M.J., Higgins J.P.T., Sterne J.A.C., Higgins J.P.T., Thomas J., Chandler J., Cumpston M., Li T., Page M.J., Welch V.A. (2019). Chapter 13: Assessing risk of bias due to missing results in a synthesis. Cochrane Handbook for Systematic Reviews of Interventions Version 60.

[121] Olmedilla-Alonso B., Granado-Lorencio F., Blanco-Navarro I. (2005). Carotenoids, retinol and tocopherols in blood: Comparability between serum and plasma (Li-heparin) values. Clin. Biochem..

[122] Kamycheva E., Berg V., Jorde R. (2013). Insulin-like growth factor I, growth hormone, and insulin sensitivity: The effects of a one-year cholecalciferol supplementation in middle-aged overweight and obese subjects. Endocrine.

[123] Gee J. (2013). Phase II Open Label, Multi-Center Clinical Trial of Modulation of Intermediate Endpoint Biomarkers by 1α-Hydroxyvitamin D2 in Patients With Clinically Localized Prostate Cancer and High Grade Pin. Prostate.

[124] Sinha-Hikim I., Duran P., Shen R., Lee M., Friedman T.C., Davidson M.B. (2015). Effect of long term vitamin D supplementation on biomarkers of inflammation in Latino and African-American subjects with pre-diabetes and hypovitaminosis D. Horm. Metab. Res..

[125] Mason C., Tapsoba J.D.D., Duggan C., Imayama I., Wang C.-Y., Korde L.A., Stanczyk F., McTiernan A. (2016). Effects of vitamin D supplementation during weight loss on sex hormones in postmenopausal women. Menopause.

[126] Zhang R.H., Chen K.J., Lu D.X., Zhu X.F., Ma X.C. (2005). A clinical study of Yigu capsule in treating postmenopausal osteoporosis. Chin. J. Integr. Med..

[127] Heijboer A.C., Oosterwerff M., Schroten N.F., Eekhoff E.M., Chel V.G., De Boer R.A., Blankenstein M., Lips P. (2015). Vitamin D supplementation and testosterone concentrations in male human subjects. Clin. Endocrinol..

[128] Lerchbaum E., Trummer C., Theiler-Schwetz V., Kollmann M., Wölfler M., Heijboer A.C., Pilz S., Obermayer-Pietsch B. (2018). Effects of vitamin D supplementation on androgens in men with low testosterone levels: A randomized controlled trial. Eur. J. Nutr..

[129] Zittermann A., Ernst J.B., Prokop S., Fuchs U., Dreier J., Kuhn J., Knabbe C., Berthold H., Gouni-Berthold I., Gummert J.F. (2019). Vitamin D supplementation does not prevent the testosterone decline in males with advanced heart failure: The EVITA trial. Eur. J. Nutr..

[130] Bonjour J.P., Benoit V., Pourchaire O., Rousseau B., Souberbielle J.C. (2011). Nutritional approach for inhibiting bone resorption in institutionalized elderly women with vitamin D insufficiency and high prevalence of fracture. J. Nutr. Health Aging.

[131] Trummer C., Theiler-Schwetz V., Pandis M., Grübler M.R., Verheyen N., Gaksch M., Zittermann A., März W., Aberer F., Lang A. (2017). Effects of Vitamin D Supplementation on IGF-1 and Calcitriol: A Randomized-Controlled Trial. Nutrients.

[132] Persson M., Hytter-Landahl A., Brismar K., Cederholm T. (2007). Nutritional supplementation and dietary advice in geriatric patients at risk of malnutrition. Clin. Nutr..

[133] Holick M.F., Lamb J., Lerman R.H., Konda V.R., Darland G., Minich D.M., Desai A., Chen T., Austin M., Kornberg J. (2010). Hop rho iso-alpha acids, berberine, vitamin D3 and vitamin K1 favorably impact biomarkers of bone turnover in postmenopausal women in a 14-week trial. J. Bone Miner. Metab..

[134] Lamb J., Holick M.F., Lerman R.H., Konda V.R., Minich D.M., Desai A., Chen T., Austin M., Kornberg J., Chang J.-L. (2011). Nutritional supplementation of hop rho iso-alpha acids, berberine, vitamin D(3), and vitamin K(1) produces a favorable bone biomarker profile supporting healthy bone metabolism in postmenopausal women with metabolic syndrome. Nutr. Res..

[135] Alehagen U., Johansson P., Aaseth J., Alexander J., Brismar K. (2017). Increase in insulin-like growth factor 1 (IGF-1) and insulin-like growth factor binding protein 1 after supplementation with selenium and coenzyme Q10. A prospective randomized double-blind placebo-controlled trial among elderly Swedish citizens. PLoS ONE.

[136] Torbergsen A.C., Watne L.O., Frihagen F., Wyller T.B., Mowe M. (2019). Effects of nutritional intervention upon bone turnover in elderly hip fracture patients. Randomized controlled trial. Clin. Nutr. ESPEN.

[137] Jensen C., Holloway L., Block G., Spiller G., Gildengorin G., Gunderson E., Butterfield G., Marcus R. (2002). Long-term effects of nutrient intervention on markers of bone remodeling and calciotropic hormones in late-postmenopausal women. Am. J. Clin. Nutr..

[138] Ranganathan P., Pramesh C.S., Aggarwal R. (2016). Common pitfalls in statistical analysis: Intention-to-treat versus per-protocol analysis. Perspect. Clin. Res..

[139] Hoenjet K.M., Dagnelie P.C., Delaere K.P., Wijckmans N.E., Zambon J.V., Oosterhof G.O. (2005). Effect of a nutritional supplement containing vitamin E, selenium, vitamin c and coenzyme Q10 on serum PSA in patients with hormonally untreated carcinoma of the prostate: A randomised placebo-controlled study. Eur. Urol..

[140] Kranse R., Dagnelie P.C., Van Kemenade M.C., De Jong F.H., Blom J.H., Tijburg L.B., Weststrate J.A., Schröder F.H. (2005). Dietary intervention in prostate cancer patients: PSA response in a randomized double-blind placebo-controlled study. Int. J. Cancer.

[141] Vidlar A., Vostálová J., Ulrichová J., Student V., Krajicek M., Vrbkova J., Simanek V. (2010). The safety and efficacy of a silymarin and selenium combination in men after radical prostatectomy-a six month placebo-controlled double-blind clinical trial. Biomed. Pap..

[142] Vostalova J., Vidlar A., Ulrichova J., Vrbkova J., Simanek V., Student V. (2013). Use of selenium-silymarin mix reduces lower urinary tract symptoms and prostate specific antigen in men. Phytomedicine.

[143] Van Amsterdam J., van der Horst-Graat J., Bischoff E., Steerenberg P., Opperhuizen A., Schouten E. (2005). The effect of vitamin E supplementation on serum DHEA and neopterin levels in elderly subjects. Int. J. Vitam. Nutr. Res..

[144] Zhu K., Meng R., A Kerr D., Devine A., Solah V., Binns C.W., Prince R.L. (2011). The effects of a two-year randomized, controlled trial of whey protein supplementation on bone structure, IGF-1, and urinary calcium excretion in older postmenopausal women. J. Bone Miner. Res..

[145] Larouche D., Hanna M., Chang S.L., Jacob S., Têtu B., Diorio C. (2017). Evaluation of Antioxidant Intakes in Relation to Inflammatory Markers Expression within the Normal Breast Tissue of Breast Cancer Patients. Integr. Cancer Ther..

[146] Watts E.L., Appleby P.N., Albanes D., Black A., Chan J.M., Chen C., Cirillo P.M., Cohn B.A., Cook M.B., Donovan J.L. (2017). Circulating sex hormones in relation to anthropometric, sociodemographic and behavioural factors in an international dataset of 12,300 men. PLoS ONE.

[147] Watts E.L., Perez-Cornago A., Appleby P.N., Albanes D., Ardanaz E., Black A., Bueno-De-Mesquita H.B., Chan J.M., Chen C., Chubb S.P. (2019). The associations of anthropometric, behavioural and sociodemographic factors with circulating concentrations of IGF-I, IGF-II, IGFBP-1, IGFBP-2 and IGFBP-3 in a pooled analysis of 16,024 men from 22 studies. Int. J. Cancer.

[148] Bingham S.A., Cassidy A., Cole T.J., Welch A., Runswick S.A., Black A.E., Thurnham D., Bates C., Khaw K.T., Key T.J.A. (1995). Validation of weighed records and other methods of dietary assessment using the 24 h urine nitrogen technique and other biological markers. Br. J. Nutr..

[149] A Bingham S., Luben R.N., Welch A., Low Y.L., Khaw K.T., Wareham N., Day N. (2008). Associations between dietary methods and biomarkers, and between fruits and vegetables and risk of ischaemic heart disease, in the EPIC Norfolk Cohort Study. Int. J. Epidemiol..

[150] Hayhoe R.P.G., Lentjes M.A.H., Mulligan A.A., Luben R.N., Khaw K.T., Welch A.A. (2017). Carotenoid dietary intakes and plasma concentrations are associated with heel bone ultrasound attenuation and osteoporotic fracture risk in the European Prospective Investigation into Cancer and Nutrition (EPIC)-Norfolk cohort. Br. J. Nutr..

