# Supplementary material for: Effects of Dietary or Supplementary Micronutrients on Sex Hormones and IGF-1 in Middle and Older Age: A Systematic Review and Meta-Analysis
Source: Nutrients. 2020 May 18;12(5):1457. doi: 10.3390/nu12051457 (PMC7284480; doi:10.3390/nu12051457)
Supplement: Supplementary file 1 [file nutrients-12-01457-s001.zip › nutrients--757773-supplementary/Supplementary Table 1A - Search Strategy Form.pdf]

Supplementary Table A – Search Strategy

Medline:

1.((diet* or nutrition* or intake* or nutrient*) adj5 (mineral* or Magnesium or Mg2+ or Zinc or Zn2+ or Selenium or Se or Potassium or K+ or Iron or Fe2+ or Fe3+ or Copper or Cu2+ or Cu3+ or Mg+)).tw.
2. (Vitamin A or Retinol* or Retinal* or Retinoic acid or Vitamin A1 or Vitamin A Aldehyde).tw.
3. exp micronutrients/
4. (Vitamin C or Ascorbic Acid or L-Ascorbic Acid or Ascorbate).tw.
5. (Vitamin D or Calciferol or Vitamin D2 or Ergocalciferol or ergosterol or vitamin D3 or cholecalciferol or 7-dehydrocholesterol or previtamin D3 or 25-hydroxycholecalciferol or calcitriol or 1,25 dihydroxycholeciferol or Calctroic acid or Vitamin D4 or dihydroergocalciferol).tw.
6. (Vitamin E or Tocopherol* or Tocotrienol* or Tocofersolan*).tw.
7. (carotenoid* or carotene* or Cryptoxanthin* or Lutein or Lycopene or Zeaxanthin).tw.
8. exp xanthophylls/
9. 1 or 2 or 3 or 4 or 5 or 6 or 7 or 8
10. (sex hormone* or Sex steroid* or Androgen* or Androstenediol or dihydrotestosterone or testosterone or androstenedione or Oestrogen* or estrogen* or Estetrol or oestetrol or Estrone or oestrone or estradiol or oestradiol or estriol or Oestriol or dehydroepiandrosterone or DHEAS or Sex hormone binding globulin or SHBG or Gonadal steroid hormone*).tw.
11. (IGF-1 or IGF-I or IGF-A or IGFI or MGF or Insulin like growth factor 1 or somatomedin C or Sulfation factor or sulphation factor).tw.
12. exp Insulin-Like Growth Factor I/
13. exp Gonadal Steroid Hormones/
14. 10 or 11 or 12 or 13
15. 9 and 14
16. (animals not (human and animals)).sh.
17. 15 not 16
18. adult/ or exp aged/ or exp institutionalized adult/ or exp middle aged/ or exp young adult/
19. 17 and 18
20. Randomized Controlled Trials as Topic/
21. randomized controlled trial/
22. Random Allocation/
23. Double Blind Method/
24. Single Blind Method/
25. clinical trial/
26. clinical trial, phase i.pt.
27. clinical trial, phase ii.pt.
28. clinical trial, phase iii.pt.
29. clinical trial, phase iv.pt.
30. controlled clinical trial.pt.
31. randomized controlled trial.pt.
32. multicenter study.pt.
33. clinical trial.pt.
34. exp Clinical Trials as topic/
35. or/20-34

36. (clinical adj trial$).tw.
37. ((singl$ or doubl$ or treb$ or tripl$) adj (blind$3 or mask$3)).tw.
38. PLACEBOS/
39. placebo$.tw.
40. randomly allocated.tw.
41. (allocated adj2 random$).tw.
42. or/36-41
43. 35 or 42
44. case report.tw.
45. letter/
46. historical article/
47. or/44-46
48. 43 not 47

Embase:

1. ((diet* or nutrition* or intake* or nutrient*) adj5 (mineral* or Magnesium or Mg2+ or
Zinc or Zn2+
or Selenium or Se or Potassium or K+ or Iron or Fe2+ or Fe3+ or Copper or Cu2+ or Cu3+ or Mg+)).tw.
2. (Vitamin A or Retinol* or Retinal* or Retinoic acid or Vitamin A1 or Vitamin A Aldehyde).tw.
3. exp micronutrients/
4. (Vitamin C or Ascorbic Acid or L-Ascorbic Acid or Ascorbate).tw.
5. (Vitamin D or Calciferol or Vitamin D2 or Ergocalciferol or ergosterol or vitamin D3 or cholecalciferol or 7-dehydrocholesterol or previtamin D3 or 25-hydroxycholecalciferol or calcitriol or 1,25 dihydroxycholeciferol or Calctroic acid or Vitamin D4 or dihydroergocalciferol).tw.
6. (Vitamin E or Tocopherol* or Tocotrienol* or Tocofersolan*).tw.
7. (carotenoid* or carotene* or Cryptoxanthin* or Lutein or Lycopene or Zeaxanthin).tw.
8. exp xanthophylls/
9. 1 or 2 or 3 or 4 or 5 or 6 or 7 or 8
10. (sex hormone* or Sex steroid* or Androgen* or Androstenediol or dihydrotestosterone or testosterone or androstenedione or Oestrogen* or estrogen* or Estetrol or oestetrol or Estrone or oestrone or estradiol or oestradiol or estriol or Oestriol or dehydroepiandrosterone or DHEAS or Sex hormone binding globulin or SHBG or Gonadal steroid hormone*).tw.
11. (IGF-1 or IGF-I or IGF-A or IGFI or MGF or Insulin like growth factor 1 or somatomedin C or Sulfation factor or sulphation factor).tw.
12. exp insulin like growth factor 1/
13. exp Sex hormone/
14. 10 or 11 or 12 or 13
15. 9 and 14
16. (animals not (human and animals)).sh.
17. 15 not 16
18. exp adult/
19. 17 and 18
20. Clinical Trial/
21. Randomized Controlled Trial/
22. controlled clinical trial/
23. multicenter study/

24. Phase 3 clinical trial/
25. Phase 4 clinical trial/
26. exp RANDOMIZATION/
27. Single Blind Procedure/
28. Double Blind Procedure/
29. Crossover Procedure/
30. PLACEBO/
31. randomi?ed controlled trial$.tw.
32. rct.tw.
33. (random$ adj2 allocat$).tw.
34. single blind$.tw.
35. double blind$.tw.
36. ((treble or triple) adj blind$).tw.
37. placebo$.tw.
38. Prospective Study/
39. or/20-38
40. Case Study/
41. case report.tw.
42. abstract report/ or letter/
43. Conference proceeding.pt.
44. Conference abstract.pt.
45. Editorial.pt.
46. Letter.pt.
47. Note.pt.
48. or/40-47
49. 39 not 48
50. 19 and 49

Cochrane:

1. ((diet* or nutrition* or intake* or nutrient*) near/5 (mineral* or Magnesium or Mg or Zinc or Zn or Selenium or Se or Potassium or K or Iron or Fe or Copper or Cu)):kw (Word variations have been searched)
2. (Vitamin A or Retinol* or Retinal* or Retinoic acid or Vitamin A1 or Vitamin A Aldehyde):kw (Word variations have been searched)
3. MeSH Descriptor: [Micronutrients] explode all trees
4. (Vitamin C or Ascorbic Acid or L-Ascorbic Acid or Ascorbate):kw (Word variations have been searched)
5. (Vitamin D or Calciferol or Vitamin D2 or Ergocalciferol or ergosterol or vitamin D3 or cholecalciferol or 7-dehydrocholesterol or previtamin D3 or 25-hydroxycholecalciferol or calcitriol or 1,25 dihydroxycholeciferol or Calcitroic acid or vitamin D4 or dihydroergocalciferol):kw (word variations have been searched)
6. (Vitamin E or Tocopherol* or Tocotrienol* or Tocofersolan*):kw (Word variations have been searched)
7. (carotenoid* or carotene* or Cryptoxanthin* or Lutein or Lycopene or Zeaxanthin):kw (Word variations have been searched)
8. MeSH descriptor: [Xanthophylls] explode all trees

9. 1 or 2 or 3 or 4 or 5 or 6 or 7 or 8
10. (sex hormone* or Sex steroid* or Androgen* or androstenediol or dihydrotestosterone or testosterone or androstenedione or Oestrogen* or estrogen* or estetrol or oestetrol or estrone or oestrone or estradiol or oestradiol or estriol or oestriol or dehydroepiandrosterone or DHEAS or sex hormone binding globulin or SHBG or gonadal steroid hormone*):kw (Word variations have been searched).
11. (IGF-1 or IGF-I or IGF-A or IGFI or MGF or Insulin like growth factor 1 or somatomedin C or sulfation factor or sulphation factor):kw (Word variations have been searched)
12. MeSH descriptor: [Insulin-like growth factor I] explode all trees
13. MeSH descriptor: [Gonadal Steroid Hormones] explode all trees
14. 10 or 11 or 12 or 13
15. 9 and 14
16. MeSH descriptor: [Animals] explode all trees
17. MeSH descriptor: [Humans] explode all trees
18. 16 not (16 and 17)
19. 15 not 18
20. adult:kw (word variations have been searched)
21. MeSH descriptor: [Aged] explode all trees
22. MeSH descriptor: [Middle aged] explode all trees
23. MeSH descriptor [Young Adult] explode all trees
24. 20 or 21 or 22 or 23
25. 19 and 24.
