# Supplementary material for: Effects of Dietary or Supplementary Micronutrients on Sex Hormones and IGF-1 in Middle and Older Age: A Systematic Review and Meta-Analysis
Source: Nutrients. 2020 May 18;12(5):1457. doi: 10.3390/nu12051457 (PMC7284480; doi:10.3390/nu12051457)
Supplement: Supplementary file 1 [file nutrients-12-01457-s001.zip › nutrients--757773-supplementary/Supplementary Table 2B - Inclusion Exclusion Form.pdf]

Supplementary Table B – Inclusion / Exclusion Form

Lead author and publication date:
Study reference number:
Name of reviewer:

**Study Design**		Yes	No	Unclear
1	Is the study described as an RCT? If yes, Complete the questions below			
2	Is the study described as a longitudinal study (cross-sectional or prospective cohort study)? If yes, Complete the questions overleaf.			
3	If the study is described as neither a RCT, Prospective cohort study or Cross-sectional study, place a cross in the box.	☐		

RCT only

**Eligibility for RCTs**		**Yes**	**No**	**Unclear**
**1**	Does the study examine participants, either:<br>- Above the age of 45?<br>- Mean age above 45 (if range given)<br>- Have more than 75% of participants over 45 years?<br>- Include results from an age bracket of over 45 years?			
**2**	Does the study examine at least one type of the following micronutrients (in isolation or combination)<br>• Oral intake on Vitamin A (or derivative(s)) against control.<br>• Oral intake on Vitamin C against control.<br>• Oral intake on Vitamin D (any type) against control.<br>• Oral intake on Vitamin E against control<br>• Oral intake on Carotenoids (any type) against control.<br>• Oral intake of individual specific minerals (Zinc, Magnesium, selenium, K+) against control.<br>• Oral intake of iron against control.<br>• Oral intake of copper against control.			
**3**	Are the primary or secondary outcomes of the study examining one of the following listed:<br>a. Androgens: androstenediol, androstenedione, dihydrotestosterone, & testosterone.<br>b. Oestrogens: E2, estradiol, estetrol, estriol, & estrone.<br>c. Dehydroepiandrosterone (DHEAS)<br>d. Sex hormone binding globulin (SHBG)<br>e. IGF-1 or IGF-I or IGF-A or IGFI or MGF or Insulin like growth factor 1 or somatomedin C or Sulfation factor or sulphation factor			
**4**	Are participants in both treatment arms receiving <u>**exactly**</u> the same treatment except the intervention itself?<br>For example:<br>• Exercise in conjunction with intervention<br>• Supplements in conjunction with micronutrients<br>• Dietary advice in conjunction with intervention			

ELIGIBLE FOR SR ☐ **NOT ELIGIBLE FOR SR** ☐ **Unsure** ☐ **Reason:**

**5**	Are participants free from any renal disease, active dialysis[(1)], or liver disease[(2)]?			

If answered yes to ALL questions, include in SR. If answered no to one or more questions, exclude from SR and add further comments if necessary.

**Further comments**

Prospective Cohort Study

**Eligibility for Longitudinal studies**		**Yes**	**No**	**Unclear**
**1**	Does the study include participants at baseline, either:<br>- Above the age of 45?<br>- Mean age above 45 (if range given)<br>- Have more than 75% of participants over 45 years?<br>- Include results from an age bracket of over 45 years?			
**2**	Does the study assess at least one type of the following micronutrients at baseline (in isolation or combination)<br>• Oral intake on Vitamin A (or derivative(s)).<br>• Oral intake on Vitamin C.<br>• Oral intake on Vitamin D (any type).<br>• Oral intake on Vitamin E.<br>• Oral intake on Carotenoids (any type).<br>• Oral intake of individual specific minerals (Zinc, Magnesium, selenium, K+).<br>• Oral intake of iron.<br>• Oral intake of copper.<br>• Biomarkers of micronutrient intake (blood, serum, plasma, body fat, nail or hair measurements).			
**3**	Are the primary or secondary outcomes of the study examining one of the following sex hormones listed:<br>a. Androgens: androstenediol, androstenedione, dihydrotestosterone, & testosterone.<br>b. Oestrogens: E2, estradiol, estetrol, estriol, & estrone.<br>c. Dehydroepiandrosterone (DHEAS)<br>d. Sex hormone binding globulin (SHBG)<br>e. IGF-1 or IGF-I or IGF-A or IGFI or MGF or Insulin like growth factor 1 or somatomedin C or Sulfation factor or sulphation factor			
**4**	Does the study examine the participants **at baseline** for sex hormones or IGF-1?			
**5**	Are participants free from any renal disease, or active dialysis[(1)]?			

ELIGIBLE FOR SR ☐ **NOT ELIGIBLE FOR SR** ☐ **Unsure** ☐ **Reason:**

If answered yes to ALL questions, include in SR. If answered no to one or more questions, exclude

Cross-sectional Study

**Eligibility for Cross-sectional study**		**Yes**	**No**	**Unclear**
**1**	Does the study include participants aged, either:<br>- Above 45?<br>- Mean age above 45 (if range given)<br>- Have more than 75% of participants over 45 years?<br>- Include results from an age bracket of over 45 years?			
**2**	Does the study assess at least one type of the following micronutrients (in isolation or combination):<br>• Oral intake on Vitamin A (or derivative(s)).<br>• Oral intake on Vitamin C.<br>• Oral intake on Vitamin D (any type).<br>• Oral intake on Vitamin E.<br>• Oral intake on Carotenoids (any type).<br>• Oral intake of individual specific minerals (Zinc, Magnesium, selenium, K+).<br>• Oral intake of iron.<br>• Oral intake of copper.<br>• Biomarkers of micronutrient intake (blood, serum, plasma, body fat, nail or hair measurements).			
**3**	Are the primary or secondary outcomes of the study examining one of the following sex hormones listed:<br>f. Androgens: androstenediol, androstenedione, dihydrotestosterone, & testosterone.<br>g. Oestrogens: E2, estradiol, estetrol, estriol, & estrone.<br>h. Dehydroepiandrosterone (DHEAS)<br>i. Sex hormone binding globulin (SHBG)<br>j. IGF-1 or IGF-I or IGF-A or IGFI or MGF or Insulin like growth factor 1 or somatomedin C or Sulfation factor or sulphation factor			
**4**	Are participants free from any renal disease, or active dialysis[(1)]?			

from SR and add further comments if necessary.

**Further comments**

Note:

If both reviewers tick YES to all questions (based on study type) then the study will be included in SR.
If both reviewers tick NO to ANY question, the study will be excluded from the SR.
If ANY reviewer ticks NO or UNSURE to ANY question, or if there is a disagreement that cannot be rectified by discussion, a third party reviewer will be involved, and this will be recorded in the SR.

ELIGIBLE FOR SR ☐ **NOT ELIGIBLE FOR SR** ☐ **Unsure** ☐ **Reason:**

If answered yes to ALL questions, include in SR. If answered no to one or more questions, exclude from SR and add further comments if necessary.

**Further comments**

Note:

If both reviewers tick YES to all questions (based on study type) then the study will be included in SR.
If both reviewers tick NO to ANY question, the study will be excluded from the SR.
If ANY reviewer ticks NO or UNSURE to ANY question, or if there is a disagreement that cannot be rectified by discussion, a third party reviewer will be involved, and this will be recorded in the SR

1. Schmidt A, Luger A, Hörl WH. Sexual hormone abnormalities in male patients with renal failure. Nephrology Dialysis Transplantation. 2002;17(3):368-71.
2. Bianchi GP, Marchesini G, Brizi M, Rossi B, Forlani G, Boni P, et al. Nutritional effects of oral zinc supplementation in cirrhosis. Nutrition Research. 2000;20(8):1079-89.

ELIGIBLE FOR SR ☐ **NOT ELIGIBLE FOR SR** ☐ **Unsure** ☐ **Reason:**
