# Supplementary material for: Effects of Dietary or Supplementary Micronutrients on Sex Hormones and IGF-1 in Middle and Older Age: A Systematic Review and Meta-Analysis
Source: Nutrients. 2020 May 18;12(5):1457. doi: 10.3390/nu12051457 (PMC7284480; doi:10.3390/nu12051457)
Supplement: Supplementary file 1 [file nutrients-12-01457-s001.zip › nutrients--757773-supplementary/Supplementary Table 3C - Data Extraction Form.pdf]

Supplementary Table C - Sample Data Extraction Form

Characteristics of Study		Ref
**Study Id**		
**Article Title**		
Author		
Funding		
Country		
Ethics		
Aims		
Study Design		
Setting		
**Subjects**<br>Inclusion<br><br>Exclusion<br><br>Sample Size calculation		
**Dependant Variable**<br><br>Measure of sex hormone<br><br>Micronutrient		
**Independent variable**<br><br>Intervention<br><br>Control		
**Intervention**<br><br>N =<br>Age (mean)<br>Gender (M/F)<br>Ethnicity<br>BMI		
**Control**<br><br>N =<br>Age (mean)<br>Gender (M/F)<br>Ethnicity<br>BMI		
Duration of study		

Supplementary Table C - Sample Data Extraction Form

Length of Follow-up & Frequency		

Outcome Data		Ref
Type of analysis		
Outcome name		
**Conclusion**		

No of participants		Lost to follow-up	**INTERVENTION**			
M	F		Baseline (s.d)	Post INTERVENTION (s.d)	P-Value	Ref

No of participants		Lost to follow-up	**CONTROL**			
M	F		Baseline (s.d)	Post CONTROL (s.d)	P-Value	Ref
