# Supplementary material for: Effects of Dietary or Supplementary Micronutrients on Sex Hormones and IGF-1 in Middle and Older Age: A Systematic Review and Meta-Analysis
Source: Nutrients. 2020 May 18;12(5):1457. doi: 10.3390/nu12051457 (PMC7284480; doi:10.3390/nu12051457)
Supplement: Supplementary file 1 [file nutrients-12-01457-s001.zip › nutrients--757773-supplementary/Supplementary Table 4D - Systematic review Cochrane Risk of Bias Form.pdf]

Supplementary Table D - The Cochrane Collaboration's tool for assessing risk of bias in Intervention Studies

Domain	Support for judgement	Review authors' judgement
**Selection bias**		
Random sequence generation	The researcher describes methods used to generate the allocation sequence such that, an assessment can be made to determine they are comparable.	Was any selection bias present (biased allocation to interventions) as a result of a poor randomisation sequence?<br><br>**YES**<br>**NO**<br>**UNCLEAR**
Comments:		
Allocation concealment	The researcher describes the method used to conceal the allocation sequence in enough detail to determine whether intervention allocations could have been foreseen prior to, or during enrolment.	Was there any selection bias (biased allocation to interventions) based on inadequate concealment of allocations prior to assigning?<br><br>**YES**<br>**NO**<br>**UNCLEAR**
Comments:		
**Performance Bias**		
Blinding of participants and personnel. Assessment should be made for each main outcome (or class of outcomes)	The researcher describes all methods used, if any, to blind study participants and personnel from knowledge of which intervention a participant received. The research should also give information as to whether the blinding was effective.	Was there any performance bias due to awareness of allocated interventions by participants and personnel during the study?<br><br>**YES**<br>**NO**<br>**UNCLEAR**
Comments:		
**Detection Bias**		
Blinding of an outcome assessment. Assessment should be made for each main outcome (or class of outcomes)	Describe all measures used (if any) to blind outcome assessors from knowledge of which intervention a participant has received. Give information relating to whether this blinding was successful.	Was there any detection bias due to knowledge of the allocated interventions by outcome assessors?<br><br>**YES**<br>**NO**<br>**UNCLEAR**

<table>
<tr><th colspan="4">Attrition Bias</th></tr>
<tr><td colspan="2">Incomplete outcome data.
Assessments should be made for each main outcome (or class of outcomes)</td><td>Does the researcher describe the completeness of outcome data for each main outcome, including attrition and exclusions from the analysis?</td><td>Was there any attrition bias due to amount, nature or handling of incomplete outcome data?

YES
NO
UNCLEAR</td></tr>
<tr><td colspan="4">Comments:</td></tr>
<tr><th colspan="4">Reporting Bias</th></tr>
<tr><td colspan="2">Selective reporting</td><td>Does the research state how the possibility of selective outcome reporting was examined by the review authors and what the findings were?

Intention to treat or Per protocol</td><td>Was there any reporting bias due to selective outcome reporting?

YES
NO
UNCLEAR</td></tr>
<tr><td colspan="4">Comments:</td></tr>
<tr><th colspan="4">Other sources of bias:</th></tr>
<tr><td>Hormonal treatments</td><td colspan="2">Does the researcher adjust for (or consider) compatibility of control and intervention groups, such as hormonal treatments, including the following?

- Hormonal replacement therapy (HRT)[1]
- 5 alpha reductase inhibitors[2, 3]
- Adrenal cortical steroids (corticotrophin, glucocorticoids, mineralocorticoids)[4]
- Adrenal corticosteroid inhibitors[4]
-Anti-androgens[5]
- Anti-gonadotropic agents[6]
- Anti-thyroid hormones / thyroid medication[7]
- Aromatase inhibitors[8]
- Calcitonin[9]
- Oestrogen receptor antagonists / Selective oestrogen receptor modulators [10]
- Gonadotrophin releasing hormone antagonists[11, 12]
- Growth hormone receptor blockers[13]
- Growth hormones[14, 15]
- Insulin like growth factor[16]
- Parathyroid hormone and analogues[17].
- Prolactin inhibitor[18]
- Sex hormones: androgens and anabolic steroids, hormonal contraception (combined pill, progesterone only pill, IUS coil, injection, patch, creams), gonadotrophin releasing hormones, gonadotrophins, progestins, sex hormone combinations.
- Somatostatin and somatostatin analogues[19].
- Synthetic ovulation stimulants[20]</td><td>Was there any evidence of hormonal medication confounding results of the study?
This may include the researcher including results from individuals who may have: started/stopped/changed dose of existing or new hormonal medication.

YES
NO
UNCLEAR</td></tr>
</table>

Dietary influences	Does the researcher consider the impact of certain dietary components influencing sex hormones? This specifically addresses participants **changing current dietary behaviours** during the study (including: starting, stopping, increasing or decreasing particular foods).<br><br>Types of foods to consider include:<br>- Soy protein isolate[21]<br>- Licorice root[22, 23]<br>- Hemp, flax and chia seeds[24]<br>- Green tea catechins[22, 25]<br>- Chaste tree (vitex angus)[22, 26]<br>- Reishi mushroom [22, 27-29]<br>- Fenugreek[30]<br>- Saw palmetto[22]<br>- Bitter melon[31]<br>- White peony[22]<br>- ATD (1,4,6-androstatrience-3,17-dione)[32]	Was there any evidence of dietary changes in participants that may influence the results of the study? This may include the researcher including results from individuals who may have:<br>**Started, stopped, increased, or decreased** amounts of food/supplements that enhance or attenuate sex hormones.<br><br>**YES**<br><br>**NO**<br><br>**UNCLEAR**
Comments:		
Micronutrient extraction	Bias through inaccuracy of micronutrient extraction can influence the true findings of the study.<br>Common methods of micronutrient extraction in epidemiological studies, include the use of "food frequency questionnaires (FFQs)".<br>Evidence suggests that, if FFQs are administered by the interviewer they are more likely to produce stronger linear correlation coefficients when compared to reference methods. Furthermore, if the researchers include or state whether dietary supplements were used, this would further minimise bias associated with micronutrient extraction[33].<br><br>Extraction of micronutrient through blood measurement will be analysed separately (sub-group), however analysis by plasma will include coagulants and other trace elements which may skew true biochemical values.<br>However, research suggests the variability between the two methods is very small[34], and reproducibility is good in both plasma and serum[35].	Is there any evidence of bias that can be drawn from the methodology of the paper?<br><br>**YES** (measurements other than FFQs, OR measurements using FFQs with no reference to supplemental use)<br>**NO** (measurements that used FFQs, with some regard/ acknowledgement for dietary supplements<br>**UNCLEAR** (The paper does not provide enough detail to work out micronutrient extraction method).
Comments:		

Outcome measurement and bias associated with measures used.	Bias through inaccuracy of hormonal extraction can influence the true findings of the study. Measurements of sex hormones using "mass spectrometry" (gas or liquid) will convey the highest accuracy, and therefore lowest amount of bias.<br>Studies that use other (less reliable) methods of hormone extraction e.g. direct immunoassay[i] or Electro chemiluminescent assay[ii], will still be eligible in the systematic review, however the reliability of methodology will reflect in conclusions drawn from that study.	Is there any evidence of bias that can be drawn from the methodology of the paper?<br><br>**YES** (measurements other than "mass spectrometry")<br><br>**NO** (measurements that only included "mass spectrometry")<br><br>**UNCLEAR** (no mention of hormone extraction method).
Sponsorship bias	Does the researcher address the possibility of sponsorship bias through the funding of the study?	Was there any evidence of funding bias in any part of the methodology, results or conclusions?<br><br>**YES**<br><br>**NO**<br><br>**UNCLEAR**

1. BNF N. Sex Hormones https://bnf.nice.org.uk/treatment-summary/sex-hormones.html2017 [Available from: https://bnf.nice.org.uk/treatment-summary/sex-hormones.html.
2. Wilborn C, Taylor L, Poole C, Foster C, Willoughby D, Kreider R. Effects of a purported aromatase and 5alpha-reductase inhibitor on hormone profiles in college-age men. International journal of sport nutrition and exercise metabolism. 2010;20(6):457-65.
3. Traish AM, Mulgaonkar A, Giordano N. The Dark Side of 5α-Reductase Inhibitors' Therapy: Sexual Dysfunction, High Gleason Grade Prostate Cancer and Depression. Korean Journal of Urology. 2014;55(6):367-79.
4. Da Silva JAP. Sex Hormones and Glucocorticoids: Interactions with the Immune System. Annals of the New York Academy of Sciences. 1999;876(1):102-18.
5. Mowszowicz I. [Antiandrogens. Mechanisms and paradoxical effects]. Annales d'endocrinologie. 1989;50(3):189-99.
6. van Poppel H, Nilsson S. Testosterone surge: rationale for gonadotropin-releasing hormone blockers? Urology. 2008;71(6):1001-6.
7. Selva DM, Hammond GL. Thyroid hormones act indirectly to increase sex hormone-binding globulin production by liver via hepatocyte nuclear factor-4alpha. Journal of molecular endocrinology. 2009;43(1):19-27.
8. de Ronde W, de Jong FH. Aromatase inhibitors in men: effects and therapeutic options. Reproductive Biology and Endocrinology : RB&E. 2011.

9. Klotz HP, Delorme ML, Ochoa F, Aussenard C. [Sex hormones and calcitonin secretion]. La semaine des hopitaux : organe fonde par l'Association d'enseignement medical des hopitaux de Paris. 1975;51(20):1333-6.
10. Jameera Begam A, Jubie S, Nanjan MJ. Estrogen receptor agonists/antagonists in breast cancer therapy: A critical review. Bioorganic Chemistry. 2017;71(Supplement C):257-74.
11. Gustofson RL, Segars JH, Larsen FW. Ganirelix acetate causes a rapid reduction in estradiol levels without adversely affecting oocyte maturation in women pretreated with leuprolide acetate who are at risk of ovarian hyperstimulation syndrome. Human reproduction (Oxford, England). 2006;21(11):2830-7.
12. Bouchard P. GnRH antagonists: Mechanism of action studies and clinical uses in women. Contraception.46(2):141.
13. Kohn DT, Kopchick JJ. Growth hormone receptor antagonists. Minerva endocrinologica. 2002;27(4):287-98.
14. Zachmann M. Interrelations between growth hormone and sex hormones: physiology and therapeutic consequences. Hormone research. 1992;38 Suppl 1:1-8.
15. Meinhardt UJ, Ho KK. Modulation of growth hormone action by sex steroids. Clinical endocrinology. 2006;65(4):413-22.
16. Huffman J, Hoffmann C, Taylor GT. Integrating insulin-like growth factor 1 and sex hormones into neuroprotection: Implications for diabetes. World Journal of Diabetes. 2017;8(2):45-55.
17. Sahovic V, Sahovic S, Grosa E, Avdic E, Helac-Cvijetic D, Kukavica N. Correlation between parathormone and sexual hormones in patients on haemodialysis. Medical archives (Sarajevo, Bosnia and Herzegovina). 2012;66(3):177-80.
18. Nitkowska M, Tomasiuk R, Czyzyk M, Friedman A. Prolactin and sex hormones levels in males with Parkinson's disease. Acta neurologica Scandinavica. 2015;131(6):411-6.
19. Ciotta L, De Leo V, Galvani F, La Marca A, Cianci A. Endocrine and metabolic effects of octreotide, a somatostatin analogue, in lean PCOS patients with either hyperinsulinaemia or lean normoinsulinaemia. Human reproduction (Oxford, England). 1999;14(12):2951-8.
20. Adamopoulos DA, Vassilopoulos P, Kapolla N, Kontogeorgos L. The effect of clomiphene citrate on sex hormone binding globulin in normospermic and oligozoospermic men. International journal of andrology. 1981;4(6):639-45.
21. Kraemer WJ, Solomon-Hill G, Volk BM, Kupchak BR, Looney DP, Dunn-Lewis C, et al. The Effects of Soy and Whey Protein Supplementation on Acute Hormonal Responses to Resistance Exercise in Men. Journal of the American College of Nutrition. 2013;32(1):66-74.
22. Grant P, Ramasamy S. An Update on Plant Derived Anti-Androgens. International Journal of Endocrinology and Metabolism. 2012;10(2):497-502.
23. Armanini D, Bonanni G, Palermo M. Reduction of Serum Testosterone in Men by Licorice. New England Journal of Medicine. 1999.
24. Prinsloo SE, van Aswegen CH. Effect of fatty acids on estradiol and testosterone binding to whole DU-145 prostate cells. Prostaglandins, leukotrienes, and essential fatty acids. 2002;66(4):419-25.
25. Das SK, Karmakar SN. Effect of green tea (camellia sinensis l.) leaf extract on reproductive system of adult male albino rats. International Journal of Physiology, Pathophysiology and Pharmacology. 2015;7(4):178-84.
26. Nasri. S HR, A. Amin. The Effects of Vitex Agnus castus L. Extract on Gonadotrophines and Testodterone in Male Mice. Iranina Int J Sci 5(1) 2004:p.25-30.

27. Liu J, Tamura S, Kurashiki K, Shimizu K, Noda K, Konishi F, et al. Anti-androgen effects of extracts and compounds from Ganoderma lucidum. Chemistry & biodiversity. 2009;6(2):231-43.
28. Liu J, Shiono J, Shimizu K, Kukita A, Kukita T, Kondo R. Ganoderic acid DM: anti-androgenic osteoclastogenesis inhibitor. Bioorganic & medicinal chemistry letters. 2009;19(8):2154-7.
29. Fujita R, Liu J, Shimizu K, Konishi F, Noda K, Kumamoto S, et al. Anti-androgenic activities of Ganoderma lucidum. Journal of ethnopharmacology. 2005;102(1):107-12.
30. Steels E, Rao A, Vitetta L. Physiological aspects of male libido enhanced by standardized Trigonella foenum-graecum extract and mineral formulation. Phytotherapy research : PTR. 2011;25(9):1294-300.
31. Naseem MZ, Patil SR, Patil SR, Ravindra, Patil RS. Antispermatogenic and androgenic activities of Momordica charantia (Karela) in albino rats. Journal of ethnopharmacology. 1998;61(1):9-16.
32. Svensson AI. The aromatase inhibitor 1,4,6-androstatriene-3,17-dione (ATD) reduces disinhibitory behavior in intact adult male rats treated with a high dose of testosterone. Behavioural brain research. 2010;206(2):216-22.
33. Henriquez-Sanchez P, Sanchez-Villegas A, Doreste-Alonso J, Ortiz-Andrellucchi A, Pfrimer K, Serra-Majem L. Dietary assessment methods for micronutrient intake: a systematic review on vitamins. The British journal of nutrition. 2009;102 Suppl 1:S10-37.
34. Olmedilla-Alonso B, Granado-Lorencio F, Blanco-Navarro I. Carotenoids, retinol and tocopherols in blood: comparability between serum and plasma (Li-heparin) values. Clinical biochemistry. 2005;38(5):444-9.
35. Yu Z, Kastenmuller G, He Y, Belcredi P, Moller G, Prehn C, et al. Differences between human plasma and serum metabolite profiles. PloS one. 2011;6(7):e21230.
