# Supplementary material for: Effects of Dietary or Supplementary Micronutrients on Sex Hormones and IGF-1 in Middle and Older Age: A Systematic Review and Meta-Analysis
Source: Nutrients. 2020 May 18;12(5):1457. doi: 10.3390/nu12051457 (PMC7284480; doi:10.3390/nu12051457)
Supplement: Supplementary file 1 [file nutrients-12-01457-s001.zip › nutrients--757773-supplementary/Supplementary Table 5E - Characteristics of RCTs_19_5_20_correct.pdf]

Supplementary Table E – Table of Characteristics for RCTs (n=26)

Author, Year (Country) [number]	Study length	Aims	Setting	Participants	Intervention	Control	Outcomes		Funding
**Alehagen, 2017 (Sweden) [135]**	4-year RCT.	To examine the effects of supplementation with selenium and coenzyme Q10 on concentrations of IGF-1 and its binding protein IGFBP-1.	Community based.	215 participants, a significant proportion had cardiovascular disease and/or metabolic dysfunction (mean age: 76.6, 53% males) were selected.	Dietary supplementation of 200 mg/day of coenzyme Q10 capsules and **200 µg/day of organic selenium yeast tablets**.	Placebo.	**IGF-1** measured by radioimmunoassay.	**Selenium** measured in blood (unclear technique).	Family Erling-Persson Foundation, Medical Research Council, Pharma Nord Aps and the County Council of OÈstergoÈtland, LinkoÈping University.
**Amsterdam, 2005 (Netherlands) [143]**	15 month RCT.	To investigate the effects of 200mg/day of Vitamin E in elderly subjects on neopterin and DHEA-S.	Community based.	100 healthy participants (mean age: 74; 50% male; BMI: 26.9) from the Netherlands area.	**Vitamin E** - Oral 200mg. 2 capsules/day for up to 15months.	Soybean oil.	**DHEAS** measured by enzyme immunoassay.	**Vitamin E** measured by "blood sample".	Unclear.
**Bonjour, 2011 (France) [130]**	6-week Cross-over trial.	To investigate the beneficial effect of the consumption of soft plain cheese on bone resorption markers in institutionalized elderly women.	Six French nursing homes or other institutions for the elderly	29 females (mean age: 86.9, BMI: 29.0) were selected.	Two servings of 100g of Soft plain cheese each were taken every day during 6 weeks. they provided daily: 164 Kcal; **2.5 µg vitamin D**; 302 mg calcium; 233 mg	Cross-over trial.	**IGF-1** measured by immunoradiometric assay.	**Vitamin D** measured by radioimmunoassay.	YOPLAIT France.

					phosphorus 14.2 g proteins.				
**Gann, 2015 (USA) [82]**	6 month RCT.	A Phase II repeat biopsy trial in men with high-grade prostatic intraepithelial neoplasia who were randomly assigned to either a placebo or capsules of 'Lyco-Mato' for a period of approximately 6 months.	North-western Memorial Hospital and the Jesse Brown Veterans Administration Medical Centre in Chicago.	58 males (mean age: 65) with a confirmed diagnosis of high grade intraepithelial prostate neoplasia.	The lycopene group consumed 2 daily capsules of Lyc-O-Mato, containing **15 mg of lycopene** per soft gel capsule ('Lycored', Ltd, Beer Sheva, Israel). The men were instructed to take 1 capsule with breakfast and 1 with dinner.	Placebo soft gel capsules contained medium-chain triglycerides and red food colouring.	**Serum IGF-1, IGFBP3** were measured by enzyme immunoassay.	**Serum lycopene** concentrations were measured by LC-MS-MS	National Institute of Health, National Institute of Cancer.
**Gee, 2013 (USA) [123]**	4 week - Phase II open label, RCT.	To determine if 1α-OH-D2 modulates intermediate endpoint biomarkers of potential significance in the development of prostate cancer.	Multi-centre trial conducted at University of Wisconsin Carbone Cancer Centre, the Medical College of Wisconsin, and the University of Iowa.	31 white males (mean age: 58.9, BMI: 29.1) with histological confirmation of prostate cancer.	**10µg/day 1α-hydroxyvitamin D2** × 4 weeks prior to surgery.	Observation only.	**IGF-1** measured by immunoassay.	Unknown measurement method for **Vitamin D metabolites.**	National Institute of Health.
**Heijboer, 2015 (Netherlands) [127]**	Post-hoc analysis of RCT.	To investigate the existence of a causal link between vitamin D and	Study 1: Heart failure patients (6 week trial).	92 males (median age: 63; median BMI: 27)	**2000 IU cholecalciferol** daily for 6 weeks. Blood was drawn in	Placebo.	**Testosterone** measured using a 2nd generation immunoassay.	**Serum 1,25(OH)2D** using liquid chromatogra	Grants from 3 independent bodies.

		testosterone. We studied the effect of vitamin D supplementation on testosterone concentrations in three independent intervention studies including males suffering from heart failure, male elderly home nursing residents, and male non-Western immigrants.	Study 2: male nursing home residents (16 week trial).<br><br>Study 3: Male non-Western Immigrants in Netherlands (16 week trial).	suffering from heart failure.	the morning, at 0, 3 and 6 weeks after starting supplements.			phy mass-spectrometry.	
				49 males (mean age: 82) were selected from a nursing home.	**Oral vitamin D3** either 600 IU per day, 4200 IU per week or 18 000 IU/month in a daily, weekly or monthly dose. In this study, only the male patients with daily supplements were studied.	Placebo.		**Serum 1,25(OH)2D** using radio-immunoassay.	ZonMw, The Netherlands Organisation for Health Research and Development & The Hague.
				42 vitamin D deficient males (mean age 53, BMI: 29) who were non-western immigrants from The Netherlands.	**1200IU of Vitamin D**. Blood was drawn, in the early morning, at baseline, after 8 and 16 weeks. All participants received calcium 500 mg per day as calcium carbonate.	Placebo.		**Serum 1,25(OH)2D** using liquid chromatography mass-spectrometry.	ZonMw, The Netherlands Organisation for Health Research and Development & The Hague.
**Hoenjet, 2005 (Netherlands) [139]**	21-week RCT.	To assess the effect of a nutritional supplement containing vitamin E,	Outpatient department.	80 males (mean age: 73.9) with a diagnosis of prostate	A daily supplement with either **vitamin E (350mg), selenium**	Placebo.	Unclear how **Testosterone was extracted.**	Unclear.	AstraZeneca, Zoetermeer, The Comprehensive Cancer Centre Limburg.

		selenium, vitamin C and coenzyme Q10 on changes in serum levels of PSA in patients with hormonally untreated carcinoma of the prostate and rising PSA levels.		cancer were included.	**(200micrograms), vitamin C (750mg), coenzyme Q10 (2x100mg)** for 21 weeks.				
**Holick, 2010 (USA) [133]**	14 week RCT.	To investigate whether supplementation with phytochemicals and nutrients essential for bone health added to lifestyle modification influences markers of bone turnover in healthy postmenopausal women.	Functional Medicine Research Centre in Gig Harbor, WA.	32 majority Caucasian post-menopausal females (mean age: 58.1, BMI: 27) with and without metabolic syndrome were selected.	1 tablet twice daily. Each tablet contained **200 mg RIAA, 100 mg berberine sulphate trihydrate (equivalent to 69 mg berberine), 500 IU vitamin D3 and 500 µg vitamin K1** + control.	Mediterranean-style, low glycaemic load diet and to engage in exercise.	**IGF-1** measured using enzyme-linked immunosorbent assay (ELISA).	**Serum vitamin D** measured by mass spectrometry.	Metagenics Inc.
**Jensen, 2002 (USA) [137]**	3-year RCT.	Comparing the long-term effects on bone turnover markers and calciotropic hormones of a	University of California.	57 postmenopausal females (mean age: 66, BMI: 25.5) were selected.	Women in the multi-nutrient group took 6 tablets of a different supplement, containing: **Ca,**	The active control group received dietary instruction targeting the consumption of **≥800 mg Ca/d,**	**IGF-1** measured by 2-site immunoassay.	**Vitamin D** was measured by extraction of serum.	Shaklee Corporation.

		multi-nutrient supplement, a calcium and vitamin D supplement, and dietary instruction aimed at increasing calcium intake through foods. (only multi-nutrient vs placebo group used in this SR).			**P, 400 IU vitamin D, Mg, B, Zn, Mn, Cu, I, Cr, Mo, Ni, Se, Sn V, Si, biotin, folic acid, niacin, pantothenic acid, vitamin A, thiamine, riboflavin, vitamin B-6, vitamin B-12, vitamin C, vitamin E, and vitamin K** (see study for doses).	**with an ideal goal of 1450 mg/d.**			
**Kamycheva, 2013 (Norway) [122]**	12 month RCT.	To determine the relationship between vitamin D status and levels of GH and IGF-1: and whether vitamin D supplementation may cause the changes in the parameters of GH-IGF-1 axis.	Outpatient department.	318 participants (mean age: 49.2; 38% male; BMI: 34.6) split into three groups: DD, DP, PP. Also stratified by BMI (<35 or >35).	**20,000IU Vitamin D** - per capsule (2x in the DD group, or 1x in the DP group) + **500mg Calcium.**	Placebo capsule (1x in DP group or 2x in PP group) + **500mg calcium.**	**IGF-1** measured using chemiluminescent immunometric assays.	**Serum vitamin D** measured by radioimmuno assay.	Northern Norway Regional Health Authority.
**Kranse, 2005 (Netherlands) [140]**	6-week RCT crossover.	The objective of this study was to show or to exclude	Departments of Urology of the Erasmus	37 males (median age 70, and weight 81kg) with	The intervention contained: **Vitamin E**	Control supplements: margarine and drink contained	**Testosterone** measured by radioimmunoassay.	**Antioxidants** measured by mass spectrometry.	Unilever Research Laboratories.

		an effect of dietary supplement on rising prostate-specific antigen (PSA) levels.	Medical Centre and the St. Franciscus Gasthuis	confirmed rising PSA levels and no evidence of prostate cancer.	**(50mg of alpha tocopherol), Phytosterols (1.5g), Selenium (0.2mg organic selenium in 0.5g bakers yeast)**. The beverage contained: **green tea, isoflavones (100mg phytoestrogens, 60mg genistein, 40mg daidzein), Carotenoids (10mg lutein, 10mg lycopene, 10mg palm carotenoids).**	none of the intervention ingredients.			
**Kucuk, 2001 (USA) [52]**	3-week RCT.	To investigate the effect of lycopene supplementation on cancerous and benign prostate tissues and on serum levels of PSA, IGF-1, and IGFBP-3 of patients with PCa.	Unclear	26 males (mean age: 62.2). Participants were: 19% African-American, 80% Caucasian American.	4-week supply of **15 mg lycopene capsules** (Lyc-O-Mato).	Placebo.	**IGF-1** measured by ELISA.	**Lycopene** measured by HPLC.	Virtual Discovery Grant from Barbara Ann Karmanos Cancer Institute, Detroit, MI.

**Lamb, 2011 (USA) [134]**	14-week RCT.	The objective of this study was to investigate whether phytochemicals and nutrients would affect the markers of bone turnover in postmenopausal women with metabolic syndrome.	Functional Medicine Research Centre in Gig Harbor, WA.	47 females (mean age: 60.3, BMI: 32.2) with metabolic syndrome were selected.	1 tablet twice daily. Each tablet contained **200 mg RIAA, 100 mg berberine sulphate trihydrate (equivalent to 69 mg berberine), 500 IU vitamin D3 and 500 µg vitamin K1**. Neither arm received supplemental calcium + control.	Mediterranean-style, low glycaemic load diet and to engage in exercise.	**IGF-1** measured by immunosorbent assay.	**Serum 25OHD measured by** liquid chromatography-tandem mass spectrometry.	Metagenics Inc.
**Lerchbaum, 2018 (Austria) [128]**	Post-hoc analysis of 12 week RCT.	To analyse the effect of vitamin D on androgen levels in men with low baseline serum total testosterone levels.	Medical University of Graz, Austria.	94 men (median age: 49, median BMI: 28.6).	Oral dose of **20,000IU** (equivalent to 2857IU/day) **vitamin D** weekly as 50 oily drops for 12 weeks.	50 oily drops without vitamin D for 12 weeks. The oil contained the same oil as the Vitamin D3 drops.	**Total testosterone** measured by liquid chromatography.	**25(OH)D** measured by liquid chromatography.	Austrian National Bank (OeNB Jubilaeumsfonds Project 14846).
**Mason, 2016 (USA) [125]**	12 month RCT.	To Investigate the effects of vitamin D3 supplementation vs placebo on changes in sex steroid hormones among overweight	Outpatient department.	218 postmenopausal women (mean age: 59.7, BMI: 32.4). Participants were: 86.2% non-hispanic white, 6.4%	**2000IU/day oral vitamin D3** supplementation alongside the ViDA weight loss program (diet and exercise component).	Placebo (sunflower oil) and VIDA weight loss program.	**Estrone, estradiol and testosterone** measured by radioimmunoassay; **SHBG** by chemiluminescent immunoassay.	**Vitamin D** measured by chemiluminescence.	Breast Cancer Research Foundation, National Cancer Institute.

		and obese post-menopausal women with low circulating vitamin D.		non-hispanic black, 0.9% hispanic and 6.4% other.					
**Persson, 2007 (Sweden) [132]**	4-month RCT.	Effects of combined nutritional treatment of patients at risk of protein-energy malnutrition (PEM) discharged from a geriatric service were evaluated.	Outpatient department.	108 elderly participants (mean age: 85, BMI: 20.2) at risk of protein-energy malnutrition (PEM) were selected.	Increased intake of fat by full-fat milk instead of low-fat milk; cream and creme fraiche in their cooking and to eat more snacks between meals. Also prescribed a liquid supplement (Sempers, 200 ml/package) and a daily multivitamin supplement: 'Friggss' (containing: **vitamins A, E, C, B1, B2, niacin, Fe, Iodine, Cu, Ma, Se, molybdenum, Chromium**).	Brief written dietary advice.	**IGF-1** measured by radioimmunoassay.	**Serum 25 OHD** measured by radio-receptor assay. **Zinc** measured by atomic absorption photo-spectrometry.	The Swedish Research Council.

**Rodondi, 2009 (Switzerland) [61]**	4 week RCT.	To assess the effect of zinc supplements on IGF-I, bone remodelling and physical performance responses in undernourished, elderly patients.	Tertiary care geriatric hospital.	61 participants (mean age: 85; 15% male), with a mini nutritional assessment score of 17-24. 8 participants untreated.	**30mg/day Zinc** + 20g oral protein (15g whey, 5g amino acids) & **550mg Calcium.**	Same as intervention, except no Zinc.	**IGF-1 & IGF-1** binding protein-3 measured by Immunoassays.	**Zinc** measured by the Randox colorimetric method.	Norvartis Consumer Health.
**Sinha-Hikim, 2014 (USA) [124]**	12 month RCT.	To examine the effect of vitamin D supplementation on non-stressed serum concentrations of hsCRP, IL-6, PAI-1, TNF-alpha and IGF-1.	Outpatient department.	80 participants (mean age: 52; 70% female; BMI: 32.7; 86.5% Latino & 13.5% African American) diagnosed with pre-diabetes and hypovitaminosis.	A weekly dose of **vitamin D3** (determined a by formula for each participant). This dose was adjusted based on the vitamin D level.	Pre-filled syringe of placebo (medium-chained triglycerides). No dietary or supplement advice given.	**IGF-1** measured by ELISA kits.	**Serum Vitamin D** measured by high performance liquid chromatography.	National Institute of Health.
**Torbergsen, 2019 (Norway) [136]**	4-month RCT.	To examine the effect of nutrition supplementation on bone turnover markers.	Orthopaedic ward , Oslo University Hospital, Norway	Total of 71 subjects (mean age: 83, BMI: 25, 79% Female).	Supplement consisting of: 150µg Vitamin K1, **20µg vitamin D3**, 1000mg Ca, **250µg Vitamin A**, **10mg Vitamin E** and 1.2g Omega-3 fatty acids.	Usual care in the orthopaedic ward without any systematic nutritional advice/supplements.	**IGF-1** measured by ELISA.	**25 (OH) D:** Radioimmunoassay; **Vitamin E:** Radioimmunoassay.	Research fellowship grant from Clinic of Medicine, Oslo University Hospital and the Sophies Minde Ortopedi Foundation. (Grant no 187980/H10).

**Trummer, 2017 (Austria) [131]**	Post-hoc analysis of 8-week RCT.	To determine whether Vitamin D supplementation vs placebo has an effect on IGF-1 concentrations (post hoc analysis of Vitamin D RCT).	Medical university of Graz.	175 participants (mean age: 60, 49% female, BMI: 30.2) with a diagnosis of hypertension and a serum vitamin D concentration of <30ng/mL.	**2800 IU of vitamin D3** as seven oily drops per day.	matching placebo as seven oily drops per day for eight weeks.	**IGF-1** measured by chemiluminescence immunoassay.	**Serum 1,25(OH)2D** measured by Chemiluminescence immunoassays.	Unclear.
**Vidlar, 2010 (Czech Republic) [141]**	6 month RCT	To evaluate the safety & tolerability of consumption of 570 mg silymarin and 240µg selenium as a dietary supplement to be used in the tertiary prevention in patients after Radical Prostatectomy.	Department of Urology, University Hospital in Olomouc, Czech Republic.	37 males (mean age: 63.7, BMI: 28.1) that were 2-3 months post Radical Prostatectomy.	**240 µg of selenium** (as selenomethionine) and 570 mg of silymarin daily for 6 months	Placebo, instructed not to consume food rich in phenolics or make dietary or lifestyle changes during the study.	**Testosterone** measured by Immunoassay.	**Plasma selenium** measured by atomic absorption spectrometry.	Czech Ministry of Education, Youth and Sports.
**Vostalova, 2013 (Czech Republic) [142]**	6 month RCT	The aim of this double-blind, placebo controlled clinical trial was to assess the effects of a combination of selenium	Department of Urology, University Hospital in Olomouc, Czech Republic.	55 healthy males (mean age: 55, BMI: 28) with lower urinary tract symptoms and benign prostate hyperplasia.	**240 µg of selenium** (in the form of yeast l-selenomethionine) plus 570 mg silymarin daily for 6 months.	Instructed not to change diet or lifestyle during the study.	**Testosterone** measured by Immunoassay.	**Plasma Selenium** measured by atomic absorption spectrometry.	Institutional Support of Palacky University in Olomouc.

		and silymarin in men with lower urinary tract symptoms, benign prostatic hyperplasia and a prostate specific antigen (PSA) ≤2.5 ng/ml							
**Vrieling, 2007 (Netherlands) [81]**	8 week RCT - crossover	To investigate the effect of supplementation with tomato-derived lycopene (30 mg/d) on serum concentrations of total IGF-I, IGF-II, IGFBP-1, IGFBP-2, and IGFBP-3.	4 hospitals in the Netherlands area.	40 males (mean age: 58.1, BMI: 26.5) and 31 females (mean age: 61.1, BMI: 27.2) were selected.	The lycopene capsules ('Lyc-OMato') contained **~15 mg total lycopene/capsule.** They were asked to take 2 capsules per day.	Placebo	**IGF-1** measured by electrochemiluminescence	**Lycopene** measured by High-performance liquid chromatography	Dutch Cancer Society.
**Walfisch, 2007 (Israel) [80]**	2-65 day RCT.	To compare concentrations of IGF-I and its binding protein IGFBP-3 in patients with colon cancer who were supplemented for a short term either	Outpatient department.	56 participants with colon cancer in the community (mean age: 68.5, 63% male, BMI: 27.35) were selected a few days-weeks before surgery.	Supplement of Lycopene containing **15 mg lycopene**. The participants were asked to take one capsule twice daily **(30mg lycopene/day)** with meals and	The placebo contained refined regular edible oil instead of the oleoresin component.	**IGF-1** measured by radioimmunoassay.	**Lycopene** measured by HPLC.	'LycoRed' Natural Product Industries.

		with a placebo or with a supplement containing lycopene.			not to change their regular diet.				
**Zhang, 2005 (China) [126]**	6 month RCT.	To evaluate the therapeutic efficacy of 'Yigu Capsule' in treating postmenopausal osteoporosis (PMO).	Unclear.	126 Asian post-menopausal females (mean age: 61.6; BMI: 22.5) that suffered from osteoporosis. Participants were split into 3 groups, 2 of which, relevant to this SR.	**Calciferol** (alpha-D3) tablet with 1 placebo capsule (no dose provided).	Placebo capsule (provided by Jinan University hospital). 4 capsules after each meal, three times daily.	Unknown measurement method for **Estradiol (E2) & Serum testosterone.**	Unknown measurement method for **calciferol.**	Natural Science Foundation of Guangdong Province (no.990475).

**Zittermann, 2018 (Germany) [129]**	Post-hoc analysis of 36 month RCT.	To investigate (via secondary analysis of the EVITA trial) whether daily vitamin D3 for 3 years is able to improve male sex hormone concentrations in patients with advanced heart failure and low vitamin D concentrations (<75nmol/l).	Outpatient department.	133 male chronic heart failure participants with low vitamin D (<75nmol/l) (mean age: 53, BMI: 29).	4000IU of cholecalciferol per day as oily drops for 3 years. Subjects remained on heart failure medication.	Matched placebo for 3 years. Medication remained the same throughout this period.	**Sex hormone binding globulin** and **total testosterone** measured by 'Architect Autoanalyzer' (Abbott, Wiesbaden, Germany).	Circulating total **25OHD, total 1,25(OH)2 D**, concentrations measured by the autoanalyzer Liaison (DiaSorin, Stillwater, MN, USA).	Sponsored by the Heart and Diabetes Center (Germany).<br><br>The Friede Springer Herz Stiftung & Merck KGaA provided funding for the study.
